# Reporting biases in self-assessed physical and cognitive health status of older Europeans

**DOI:** 10.1371/journal.pone.0223526

**Published:** 2019-10-08

**Authors:** Sonja Spitzer, Daniela Weber

**Affiliations:** 1 World Population Program at the International Institute for Applied Systems Analysis (IIASA), Wittgenstein Centre for Demography and Global Human Capital, Laxenburg, Austria; 2 Health Economics and Policy Division of the Vienna University of Economics and Business, Vienna, Austria; Universitá degli Studi di Bergamo, ITALY

## Abstract

This paper explores which demographic characteristics substantially bias self-reported physical and cognitive health status of older Europeans. The analysis utilises micro-data for 19 European countries from the Survey of Health, Ageing and Retirement in Europe to compare performance-tested outcomes of mobility and memory with their self-reported equivalents. Relative importance analysis based on multinomial logistic regressions shows that the bias in self-reported health is mostly due to reporting heterogeneities between countries and age groups, whereas gender contributes little to the discrepancy. Concordance of mobility and cognition measures is highly related; however, differences in reporting behaviour due to education and cultural background have a larger impact on self-assessed memory than on self-assessed mobility. Southern as well as Central and Eastern Europeans are much more likely to misreport their physical and cognitive abilities than Northern and Western Europeans. Overall, our results suggest that comparisons of self-reported health between countries and age groups are prone to significant biases, whereas comparisons between genders are credible for most European countries. These findings are crucial given that self-assessed data are often the only information available to researchers and policymakers when asking health-related questions.

## Introduction

Understanding the bias in self-reported health and its determinants is of utmost importance, because subjective data are often the only information at hand when researchers and policymakers ask health-related questions. These data are readily available as their collection takes less time and is more cost-effective than performance-based health measures. However, several studies show discrepancies between tested and self-reported health indicators [1–9]. In a meta-analysis, [1] find that correlation coefficients of tested and self-reported functional ability range from -0.72 to 0.60. Thus, subjective health measures are prone to bias. Assuming an underlying true but unobservable health status, survey respondents will report a higher or lower level of health depending on their demographic characteristics. Over- and underestimating health does not only harm the reliability of survey data, but also individuals themselves. Overrating health, for example, is associated with riskier health behaviour. Older individuals that overestimate their physical ability are more prone to suffer fall-induced injuries [10].

Research analysing the reporting bias in subjective health is growing and can be categorised into three streams based on the methods applied. A common strategy is to analyse the determinants of and variation in general self-rated health [11–15]. A second approach is the application of vignette methods, in which it is assumed that survey participants rate vignettes similarly to their own health [16–18]. However, there is evidence that the vignette method does not capture the full scale of reporting heterogeneity in health [16,17]. Finally, reporting biases can be evaluated directly by matching survey participants’ reports on their health with their actual tested health. In comparison with other techniques, the most important advantage of this method is that the response behaviour of each survey participant can be directly evaluated in view of his or her individual characteristics, while being fully flexible on the specification of the relationship between the tested and the self-reported variables. To date, however, this strategy has only been applied in small-scale studies evaluating either self-assessed physical health [1,2] or self-assessed cognitive abilities [3,19,20], but never both of them simultaneously.

Our scientific contribution is threefold. First, we quantify which demographic characteristics most relevantly contribute to the overall bias in subjective health. The demographic characteristics analysed in this study are those commonly used for health comparisons and thus collected in most surveys, namely country of residence, gender, age and education. To this end, we conduct a relative importance analysis allowing us to clearly identify which characteristics contribute the largest bias and consequently should not be compared based on self-reports only. To the best of our knowledge, no previous research has decomposed the bias in subjective health into its contributing determinants. Second, we directly match performance-based health measures with their self-reported equivalent for a large cross-country dataset that allows country comparisons of reporting behaviour. As a result, we can quantify the cultural bias in self-reports based on the direct comparison of objective and subjective measures, without using indirect methods such as vignettes. Third, we analyse and compare discrepancies in self-reported data for two health dimensions simultaneously, namely, self-reported physical and cognitive abilities. This allows us to explore whether the two health dimensions are correlated due to similarities in reporting style.

The analysis utilises data from the Survey of Health, Ageing and Retirement in Europe (SHARE), which comprises more than 200,000 observations of adults aged 50 to 94 from 19 European countries. We construct three-category outcome variables that indicate if an individual overestimates his or her health, underestimates his or her health, or achieves concordance between performance-tested and self-reported indicators. Multinomial logit regression allows a clear estimation of the effects of demographic characteristics on reporting behaviour. Then the relative importance of these characteristics for explaining the reporting biases is evaluated by decomposing the regression’s fit statistics. Hence, we quantify the contribution of demographic characteristics to the bias in self-reported health based on how much of the variation in concordance these characteristics explain.

Our findings show that misreporting of physical and cognitive health differs substantially between countries and age groups. The large variation in reporting style between age groups can partly be explained by differences in employment status. Educational attainment influences reporting behaviour too, especially when individuals are asked to evaluate their cognitive ability. Men and women also evaluate their health status differently, but these differences are less important in explaining the overall reporting bias. We provide a range of robustness analyses to observe whether our results are sensitive to the definition of physical and cognitive impairment, sample composition and model specifications.

The remainder of this paper is structured as follows. The dataset is introduced in Section 2 with a description of both the self-reported and performance-based variables utilised. Next, the methods used are explained in Section 3. Sections 4 and 5 present our results, which are discussed and compared with previous work in Section 6. Additional estimations along with robustness analyses are provided in S1 Appendix.

## Data and variables

The data analysed are provided by SHARE, a cross-country panel study of non-institutionalised individuals aged 50 and older who regularly live in one of the participating European countries [21–25]. The survey was launched in 2004/2005 in 11 European countries with more countries joining in the follow-up waves, resulting in 18 countries participating in 2015 in Wave 6. SHARE was reviewed and approved by the Ethics Committee of the University of Mannheim and the Ethics Council of the Max Planck Society [26].

For our analysis, we require pairs of tested and self-assessed health measures that can be matched directly. SHARE provides two such pairs, namely for mobility and cognition. Since the performance-based test for mobility is conducted in Wave 2 (2006/2007) and Wave 5 (2013) only, we pool these waves to analyse self-reports of physical health [27,28]. Wave 4 (2010–2012) and Wave 5 provide suitable data for the analysis of self-assessed cognitive health [29]. In summary, the analysis is based on pooled cross-sectional data with 88,087 observations from 17 different countries for mobility and 115,785 observations from 17 different countries for cognition.

### Outcome variables

We investigate the reporting behaviour in two health dimensions, mobility and cognition, by comparing the results of a performance test and its adequate self-report. The self-reports are requested prior to the respective performance test for mobility and cognition, and thus the test results do not influence the subjective health measures.

We assume that the performance test and its self-report cover the same health dimension. Therefore, we are able to assess whether the two variables coincide, after dichotomising them where necessary (see Subsection 2.1.2). Consequently, three different combinations of objective and subjective health measures are possible for each survey participant in the study. First, respondents achieve concordance if they have the same outcome in both the performance-tested and self-reported variable. Importantly, we do not distinguish between positive agreements (i.e. no impairment according to the test as well as the self-report) and negative agreement (i.e. impairment according to the test as well as the self-report). Second, respondents are considered to be overestimating their health if they report no impairment but are actually impaired according to the performance test. Third, respondents are considered to be underestimating their health if they report impairments but show no impairment during the performance test.

#### Mobility indicators

Performance-based mobility is measured by a chair stand test conducted in Waves 2 and 5. While all individuals were asked to perform a chair stand test in Wave 5, only individuals aged 75 years or younger were asked to do this test within Wave 2. Because Greece, Ireland, and Poland only participated in Wave 2, concordance of mobility measures can only be observed for the population aged 50–75 in these three countries.

**Fig 1:**
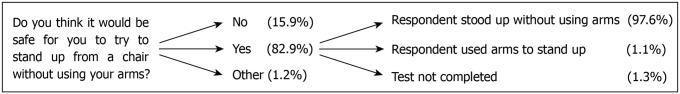
Sequence of questions and proportions of answers ascertaining tested mobility.

For the mobility performance task, survey participants were asked to stand up from a chair without using their arms. Specifically, the interviewer gave the instruction, “I would like you to fold your arms across your chest and sit so that your feet are on the floor; then stand up keeping your arms folded across your chest. Like this…”. Following this introduction, survey participants were asked whether they thought it would be safe to try standing up from a chair without using their arms (Fig 1 summarises the exact sequence of questions). Everybody completing the performance test successfully is coded as unimpaired, whereas individuals are considered impaired if they did not complete the test or if they thought it was unsafe to try in the first place. Moreover, a small percentage (1.1%) of individuals used their arms to stand up from the chair; this is also considered to be unimpaired. We provide sensitivity analyses in which individuals who thought it was unsafe to perform are excluded from the analysis, and a second set of sensitivity analyses in which individuals using their arms to stand up from the chair are considered as impaired (Tables A and B in S1 Appendix).

The self-reported mobility measure is based on the survey question, “Please tell me whether you have any difficulty doing each of the everyday activities […]. Exclude any difficulties that you expect to last less than three months”. Among other everyday activities, survey respondents could choose difficulties in “getting up from a chair after sitting for long periods”. Individuals are considered impaired if they reported having difficulties getting up from a chair.

#### Cognition indicators

Cognition was addressed with a memory test in Waves 4 to 6. Because the self-reported memory item has more than 80% missing values in Wave 6, this study only considers Waves 4 and 5.

Self-reported memory is evaluated with the survey question, “How would you rate your memory at the present time?”, which was answered on a Likert scale with categories (1) excellent, (2) very good, (3) good, (4) fair, and (5) poor. Every individual reporting fair or poor memory is considered impaired [30]. The memory performance task reports the ability to immediately recall as many words as possible. The interviewer reads aloud a list of 10 words and asks the survey participant to recall as many of the words as he or she can within 1 minute, in any order. In this study, individuals are considered to be cognitively impaired if they recall only three words or less [31,32]. Additionally, in robustness analyses, individuals are considered impaired if they recall only two or fewer words (Tables C and D in S1 Appendix). Since the subjective memory question might refer to immediate and delayed memory, we conduct an additional sensitivity analysis in which we operationalise objective cognition with delayed word recall (Table E in S1 Appendix).

### Determinants of concordance

Scientific studies on health-related questions as well as governmental health reports usually include separate analyses for one or more subpopulations. The subpopulations that are most commonly compared are individuals from different countries, different genders, age groups and educational groups. Often, these analyses are based on self-assessed health data, which is crucial since these demographic characteristics are frequently identified in the literature as important factors of health misreporting [11,13,14,16,17,33,34]. For example, [14] showed that variations in self-assessed health between European countries would be much smaller if all countries had the same reporting behaviour. These disparities are explained by cultural differences in reporting behaviour, different perceptions of how restricting poor health is and compositional differences [11]. It was also shown that older individuals often overestimate their health [35], possibly due to peer effects [36]. Some evidence suggests that women tend to underestimate their health [9], which could be related to them reporting limitations more frequently [37–39]. However, other studies find no effect of gender on reporting behaviour [15]. Finally, evidence on educational attainment shows that highly educated older Europeans are more likely to rate their health state negatively and that consequently, health inequalities appear lower than they actually are [16]. Similar results were found for non-European countries [33].

Based on the observation that demographic characteristics are most commonly used for comparative health studies, and that the same characteristics are associated with deviations in reporting behaviour, this study focuses on the main demographic characteristics only (i.e. country of residence, gender, age and educational attainment). In accordance with the International Standard Classification of Education, education levels are combined into three groups [40]. The group of low education includes everyone with lower secondary education and less. Medium education refers to survey participants with upper secondary or post-secondary non-tertiary education, and tertiary education includes individuals with tertiary education. Age is operationalised as a categorical variable, grouping 5-year age groups. Only participants between the ages 50 and 94 are considered, resulting in a total of nine age groups.

In addition to the main demographic characteristics, other individual factors such as marital status, parenthood or employment status might contribute to or mediate the effect of demographic characteristics on reporting behaviour. For example, employment status might impact health perception since persons working in analytical jobs experience their level of cognition regularly and persons conducting manual labour are likely aware of their mobility impairments. The employment status of older Europeans is highly correlated with their age, since most individuals exit the labour force at a set retirement age. Thus, parts of the effect of age on reporting behaviour might be due to differences in the employment status. Furthermore, employment might also mediate the effect of education on health perception, since highly educated individuals are more likely to work in jobs that require strong cognitive skills. While results for such subordinate channels are not presented in the main document, supplementary analyses including additional determinants are provided in S1 Appendix.

## Methods

We first investigate trends with descriptive statistics. Following this, the relationship between demographic characteristics and the probability to overestimate or underestimate health is estimated. Finally, a relative importance analysis highlights the magnitude of each explanatory variable’s contribution to the overall reporting bias. The empirical strategy employed is based on a recent study by Angel et al. [41], who analysed the reporting bias in survey-based income data. All of our analyses are first applied to indicators of mobility and then to indicators of cognition.

### Multinomial logistic regression

A multinomial logit model is applied to estimate the effects of demographic characteristics on the probability to overestimate or underestimate health. The characteristics of interest are gender, age, education, and country of residence. In addition, we control for the survey wave to account for potential time effects.

The outcome variables used in the regression models are three-category variables that indicate if an individual overestimates his or her health, underestimates his or her health, or achieves concordance between performance-tested and self-reported indicators. Concordance is used as the reference category; hence, the log odds of the variables explaining overestimating and underestimating have to be interpreted relative to the outcome category of concordance. More specifically, the non-linear baseline models are as follows:

$$\text{ln}\left(\frac{\text{P}(\text{y}=\text{over-estimating})}{\text{P}(\text{y}=\text{concordance})}\right)=\beta_{1.0}+\beta_{1.1}{\text{COUNTRY}}_{\text{i}}+\beta_{1.2}{\text{AGE}}_{\text{i}}+\beta_{1.3}{\text{EDUC}}_{\text{i}}+\beta_{1.4}{\text{GENDER}}_{\text{i}}+\beta_{1.5}{\text{WAVE}}_{\text{i}}+\varepsilon_{\text{i}}$$ (1)

$$\text{ln}\left(\frac{\text{P}(\text{y}=\text{under-estimating})}{\text{P}(\text{y}=\text{concordance})}\right)=\beta_{2.0}+\beta_{2.1}{\text{COUNTRY}}_{\text{i}}+\beta_{2.2}{\text{AGE}}_{\text{i}}+\beta_{2.3}{\text{EDUC}}_{\text{i}}+\beta_{2.4}{\text{GENDER}}_{\text{i}}+\beta_{2.5}{\text{WAVE}}_{\text{i}}+\varepsilon_{\text{i}}$$ (2)

COUNTRY_i_ is a dummy variable indicating the country of residence of each individual with the reference country being Slovenia. AGE_i_ indicates the 5-year age group of individual i with age group 60–64 as the reference category. The binary variable GENDER_i_ is 1 if the survey participant is female. EDUC_i_ is a three-category variable, and medium education serves as the reference category. WAVE_i_ is a dummy variable indicating the respective survey wave. When analysing mobility, the reference category is Wave 2; when analysing memory, the reference category is Wave 4. The standard errors are clustered at the individual level since respondents could participate in more than one wave. First, models 1 and 2 are estimated for the pooled sample including all countries. Then the models are estimated for each country separately to analyse how the effects vary by country. In the country-specific estimations, the wave dummies are only included if the respective country participated in both waves.

### Relative importance analysis

To analyse the contribution of individual characteristics to the overall bias in self-reported mobility and cognition, relative importance analysis is conducted. More specifically, the fit statistics of the regression models are decomposed to evaluate how much of the variation in concordance, overestimating, and underestimating is explained by the regressors COUNTRY_i_, AGE_i_, GENDER_i_, EDUC_i_, and WAVE_i_.

We utilise the user-written programme domin for Stata to calculate the relative contributions [42,43]. For this purpose, different models with all possible combinations of the five explanatory variables except the constant-only model are estimated. The fit statistic, in our case a Pseudo R^2^, varies depending on the constellation of the regressors. Based on this variation, the relative contribution of each explanatory variable can be computed. Importantly, only explained variation can be decomposed. Hence, only the contribution of variables actually included in the model can be quantified. We calculate the relative importance of each explanatory variable in the pooled model, as well as in the country-specific models.

### Robustness analyses

In addition to the main model specification described above, we provide robustness analyses in S1 Appendix to analyse if the results are sensitive to the definition of physical and cognitive impairment, sample composition and model specifications. First, we control for additional variables to analyse the robustness of the estimated coefficients. In particular, we add employment status, a dummy variable that indicates whether the survey participant has children, and a dummy variable that indicates whether the individual is married or in a registered partnership to the models (Tables J-O in S1 Appendix). Furthermore, education is interacted with gender to determine if the effects of education vary with gender (Tables P and Q in S1 Appendix). We also investigate whether learning effects influence the estimates. That is, when individuals had their mobility or memory tested in a previous wave, they might be more likely to achieve concordance in a subsequent wave. To control for a potential learning effect, dummy variables are added to the model, which indicate if an individual performed a test in any wave prior to the one investigated (Tables R and S in S1 Appendix).

We also analyse whether the results are sensitive to the definition of mobility impairment. In particular, we investigate whether the results change when individuals that have to use their arms to stand up from a chair are considered impaired (Table A in S1 Appendix) or when individuals that refuse standing up from a chair are dropped from the analysis (Tables A and B in S1 Appendix). We also investigate whether the results are robust to different thresholds defining memory impairment (Tables C and D in S1 Appendix). Furthermore, we use delayed word recall instead of immediate word recall to operationalise memory for a sensitivity analysis (Table E in S1 Appendix).

Finally, we investigate if the results are robust to different sample compositions. First, all frail individuals are excluded from the sample [44,45]. This allows us to account for the fact that frail individuals might be more likely to live in institutions in some countries than in other countries and consequently are not always included in our target population. These differences in sample compositions could alter the results, if poor health has an impact on reporting behaviour (Tables F and G in S1 Appendix). Second, we run the models on the exact same sample for both health dimensions. For the main analysis, Wave 2 and Wave 5 are utilised to estimate concordance of mobility measures, and Wave 4 and Wave 5 are utilised to estimate concordance of cognition measures. Since we want to compare the results for concordance of mobility and cognition measures, we also compute estimates based on Wave 5 only, which provides data for both health dimensions. Thus, we ensure that differences between the two samples are not mistakenly interpreted as differences in reporting behaviour (Tables H and I as well as Figs A and B in S1 Appendix).

## Results on mobility

### Descriptive results

When asked about their mobility, 19.2% of the survey participants report difficulties getting up from a chair after sitting for long periods. However, when tested, only 17.2% are unable to stand up from a chair or considered it unsafe to try. Overall, 80.4% of the survey participants show concordance between their reported and tested mobilities, yet the outcome varies substantially by individual characteristics. Men are more likely to report their actual level of mobility than females, mainly because women tend to more frequently underestimate their health. Interestingly, 12.0% of all women rate their mobility lower than it actually is compared to 7.9% of all men (Table 1).

**Table 1:** Summary statistics showing heterogeneities in self-reported mobility and cognition.

	Mobility						Cognition					
	Impairment		Concordance				Impairment		Concordance			
	S	T	S = T	S>T	S<T		S	T	S = T	S > T	S < T	
	%	%	%	%	%	N	%	%	%	%	%	N
**Total**	19.2	17.2	80.4	9.4	10.2	88,087	29.4	16.1	71.8	7.5	20.7	115,785
**Gender**												
Men	14.9	15.2	82.8	9.3	7.9	39,417	28.1	17	72.3	8.3	19.3	51,013
Women	22.7	18.8	78.4	9.6	12.0	48,670	30.4	15.3	71.4	6.8	21.8	64,772
**Age**												
50–54	10.3	10.0	85.5	7.1	7.4	11,229	17.6	6.3	80.6	4.0	15.4	13,244
55–59	12.7	11.6	83.9	7.5	8.5	16,196	20.5	7.1	77.9	4.3	17.7	19,461
60–64	14.9	12.5	82.3	7.6	10.0	16,836	22.9	8.7	75.4	5.2	19.4	21,098
65–69	16.6	14.7	80.2	9.0	10.8	15,721	26.5	11.3	72.9	6.0	21.1	19,447
70–74	20.7	19.5	78.0	10.5	11.5	12,906	33.8	17.0	66.9	8.2	24.9	16,180
75–79	26.9	25.0	75.8	11.7	12.5	7,347	42.0	27.6	62.2	11.8	26.0	12,350
80–84	34.4	36.7	71.4	15.9	12.7	4,664	48.5	39.3	61.4	14.9	23.7	8,525
85–89	42.6	49.8	69.1	19.5	11.4	2,438	52.3	50.0	63.5	17.4	19.1	4,283
90–94	46.9	60.2	65.6	24.7	9.7	750	53.2	55.0	63.9	19.5	16.5	1,197
**Education**												
Low	24.7	23.6	76.4	12.2	11.4	35,808	39.7	27.4	64.8	11.6	23.6	46,113
Medium	16.9	14.4	81.4	8.4	10.3	31,953	24.8	9.6	74.4	5.2	20.4	43,362
High	11.8	9.5	86.3	6.0	7.7	19,058	17.7	5.7	80.7	3.7	15.6	24,337
**Country**												
Austria	20.8	17.9	80.1	9.0	11.0	5,032	17.8	11.6	80.8	6.4	12.8	9,028
Belgium	19.5	14.1	80.8	7.4	11.9	7,932	24.4	13.5	73.8	7.7	18.5	10,511
Czechia	23.2	21.3	78.1	10.6	11.2	7,651	30.0	11.6	71.8	5.0	23.2	10,609
Denmark	12.7	7.6	87.7	4.2	8.1	6,014	17.3	9.0	81.3	5.2	13.5	6,171
Estonia	29.1	26.3	76.6	10.3	13.1	5,454	51.4	16.5	56.2	4.4	39.4	11,792
France	16.3	17.2	79.9	11.0	9.0	6,566	31.9	17.6	68.4	8.6	23.0	9,796
Germany	19.6	13.8	80.3	7.5	12.1	7,700	22.4	10.1	76.3	5.7	17.9	7,099
Greece	18.1	18.7	78.6	13.6	7.8	2,601	.	.	.	.	.	.
Hungary	.	.	.	.	.	.	34.2	17.2	67.8	7.6	24.6	2,938
Ireland	18.0	20.1	78.3	13.6	8.1	792	.	.	.	.	.	.
Italy	19.4	24.1	76.1	15.0	8.9	6,919	32.9	22.7	69.6	10.3	20.1	7,895
Luxembourg	21.2	16.1	78.8	8.3	12.9	1,561	18.5	15.5	77.4	9.9	12.6	1,543
Netherlands	14.7	10.1	85.8	5.1	9.1	6,258	15.7	10.8	80.7	7.2	12.1	6,770
Poland	29.5	29.3	70.4	17.0	12.6	1,969	32.8	24.4	69.0	11.1	19.9	1,678
Portugal	.	.	.	.	.	.	45.4	29.3	61.6	11.1	27.3	1,899
Slovenia	20.9	19.5	77.9	10.5	11.6	2,873	26.9	20.4	71.8	11.0	17.2	5,511
Spain	21.8	24.4	78.3	13.3	8.4	8,011	41.1	34.0	67.0	12.9	20.1	9,628
Sweden	15.4	10.9	83.7	6.5	9.8	6,611	29.3	12.2	71.0	6.2	22.9	6,346
Switzerland	11.2	9.3	85.6	6.6	7.9	4,143	16.5	8.2	81.6	5.2	13.3	6,571
**Wave**												
Wave 2	18.6	16.6	79.8	10.9	9.2	26,973	.	.	.	.	.	.
Wave 4	.	.	.	.	.	.	29.4	16.9	71.6	7.9	20.5	55,172
Wave 5	19.5	17.4	80.6	8.8	10.6	61,114	29.4	15.3	72.0	7.1	20.9	60,613

Note: S refers to self-reported impairment and T refers to tested impairment. S = T denotes concordance, S>T denotes overestimating, and S<T denotes underestimating. N = 100%

Concordance strongly declines with age. In the 50–54 age group, 85.5% report their correct level of mobility, but in the 90–94 age group, only 65.6% achieve concordance. Overestimating increases from 7.1% at ages 50–54 to 24.7% at ages 90–94. Underestimating increases less steeply and not linearly from 7.4% to 9.7%. There is also a clear education gradient in reporting behaviour. Highly educated individuals are more likely to achieve concordance (86.3%) than less-educated individuals (76.4%). In addition, the less educated more often overestimate their health, whereas the highly educated more often underestimate their health.

Finally, concordance varies strongly between countries. Overall, it is much higher in Northern and Western European countries than in Southern European countries, Central and Eastern European (CEE) countries, and Ireland. Denmark has the highest average concordance of 87.7%, and Poland has the lowest with only 70.4%. The variation in concordance may stem from differences in overestimating rather than underestimating, as participants from Southern and CEE countries as well as Ireland tend to strongly overestimate their mobility. Furthermore, all Southern countries are less likely to underestimate their ability to stand up from a chair.

### Regression analysis

Most findings from the descriptive analysis are confirmed by regression analyses for both the pooled sample with all countries as well as the country-specific samples (Table 2). When estimating Models 1 and 2 for the pooled sample, the coefficients show a drastic decline of concordance with age. Individuals aged 80–84 are 2.7 times more likely to overestimate their mobility than 60- to 64-year-olds (log odds 0.976). Participants aged 90–94 are 4.4 times more likely to overestimate than the reference group (log odds 1.489). The tendency to underestimate mobility also increases with age, but less strongly than the tendency to overestimate. Furthermore, underestimating peaks at ages 80–84, but decreases again for the oldest individuals. For a better overview, S1 Fig provides the predicted values of concordance based on the country-specific estimations by age group. When employment is added to the model, the age gradient in concordance remains, but appears less steep. This finding indicates that parts of the strong age effect are due to difference in the employments status between age groups (Table J in S1 Appendix).

**Table 2:** Multinomial logistic estimation for concordance of mobility measures.

	Overestimating	SE	Underestimating	SE
**Country (Ref: Slovenia)**				
Austria	-0.195*	0.080	-0.050	0.076
Belgium	-0.422***	0.077	0.083	0.071
Czechia	-0.061	0.074	-0.053	0.071
Denmark	-0.966***	0.092	-0.307***	0.079
Estonia	-0.031	0.077	0.111	0.072
France	-0.085	0.075	-0.249***	0.075
Germany	-0.299***	0.076	0.159*	0.070
Greece	0.045	0.089	-0.302**	0.098
Ireland	0.164	0.125	-0.156	0.148
Italy	0.219**	0.072	-0.280***	0.075
Luxembourg	-0.195	0.112	0.150	0.097
Netherlands	-0.864***	0.087	-0.285***	0.076
Poland	0.395***	0.092	0.303**	0.095
Spain	0.034	0.072	-0.402***	0.074
Sweden	-0.636***	0.082	-0.195**	0.074
Switzerland	-0.607***	0.090	-0.432***	0.085
**Age (Ref: 60–64)**				
50–54	-0.134**	0.048	-0.356***	0.045
55–59	-0.048	0.042	-0.179***	0.038
65–69	0.193***	0.041	0.099**	0.036
70–74	0.334***	0.042	0.156***	0.039
75–79	0.569***	0.049	0.245***	0.045
80–84	0.976***	0.053	0.301***	0.054
85–89	1.199***	0.063	0.206**	0.072
90–94	1.489***	0.096	0.092	0.132
**Women**	0.054*	0.024	0.458***	0.024
**Education (Ref: Medium)**				
Low	0.182***	0.030	0.163***	0.028
High	-0.289***	0.038	-0.299***	0.035
**Wave 5**	-0.414***	0.030	0.028	0.029
Constant	-1.965***	0.075	-2.269***	0.072
N	86,819	Pseudo R^2^		0.033

Note: The dependent variable is a three-category variable that indicates if an individual achieved concordance (reference category), overestimated or underestimated his or her health. Coefficients are given in log odds, standard errors are clustered at the individual level,

*p<0.05,

**p<0.01,

***p<0.001

Women are 1.4 times more likely to underestimate their mobility than men (log odds 0.301); in regard to overestimating, the gender effects are small (log odds 0.054) and appear insignificant once we control for employment, marriage or an interaction effect between education and gender (Tables J, N and P in S1 Appendix) as well as once participants that felt unsafe are excluded from the sample (Table B in S1 Appendix).

Similar to the descriptive results, the regression results indicate a clear education gradient in concordance. Less-educated participants are 1.2 times more likely to overestimate their mobility (log odds 0.182) and also 1.2 times more likely to underestimate their mobility (log odds 0.163) compared to individuals in the medium education group. On the contrary, participants with a tertiary education have a lower tendency to both overestimate (log odds -0.287) and underestimate mobility (log odds -0.299). There is also an interaction between gender and education, where less-educated women in particular are prone to underestimating their ability to stand up from a chair (Table P in S1 Appendix). Similarly to age, the education gradient in concordance appears less steep once employment is controlled for, which supports the hypothesis that parts of the education effect are due to educational differences in employment (Table J in S1 Appendix).

**Fig 2:**
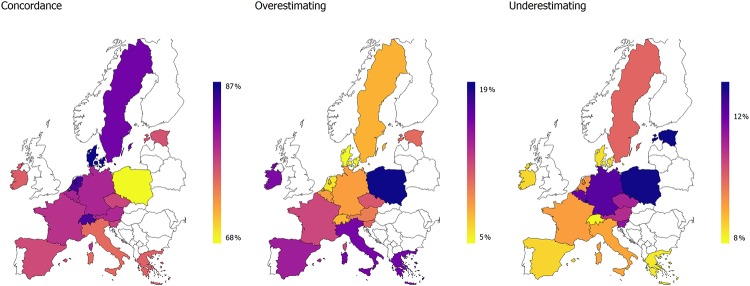
Concordance between tested and self-reported mobility by country (predicted shares).

Fig 2 presents the rates of concordance, overestimating, and underestimating by country. Overall, there is a tendency for higher concordance in Western and Northern European countries. By contrast, individuals in Southern European countries, CEE countries, and Ireland are less likely to achieve concordance, mainly because they tend to more often overestimate their mobility. The tendency to underestimate mobility is more evenly distributed among countries, yet there are still differences. For example, Southern Europeans underestimate their health less often.

Finally, the coefficient for the survey waves indicates that survey participants are less likely to overestimate their mobility in 2013 compared to 2006/2007 (log odds -0.414). The coefficient decreases after controlling for potential learning effects, but still remains significant (Table R in S1 Appendix). This could be due to cohort effects, but it is not possible to disentangle cohort effects from period effects using the present dataset. A second explanation for the significant time effects could be that some countries changed their interview procedure between the two survey waves.

When estimating models 1 and 2 for the country-specific samples, the results from the pooled model are confirmed. However, standard errors are larger due to the smaller sample sizes, leading to less significant results. The output tables for the country-specific estimations can be provided upon request. Furthermore, the results are robust to different specifications of impaired mobility (Tables A and B in S1 Appendix) as well as to different sample compositions (Tables F and H as well as Figs A and B in S1 Appendix).

### Relative importance analysis

Relative importance analysis for the pooled model shows that most of the bias in self-reported mobility stems from differences in reporting behaviour by country and age. Country differences in reporting behaviour contribute 35.0% of the explained variance in concordance, overestimating, and underestimating. Differences between age groups explain 32.1% of the bias. Together, country and age explain more than two-thirds of the variance. Reporting heterogeneity by education contribute another 17.1%, and differences by gender contribute only 11.3%. Differences by survey waves (4.6%) contribute only nominally. When employment is added to the analysis, age and education explain relatively less of the variation, which indicates again that parts of the strong age and education effects are due to differences in employment status. For additional robustness analyses, please consult S1 Appendix.

**Fig 3:**
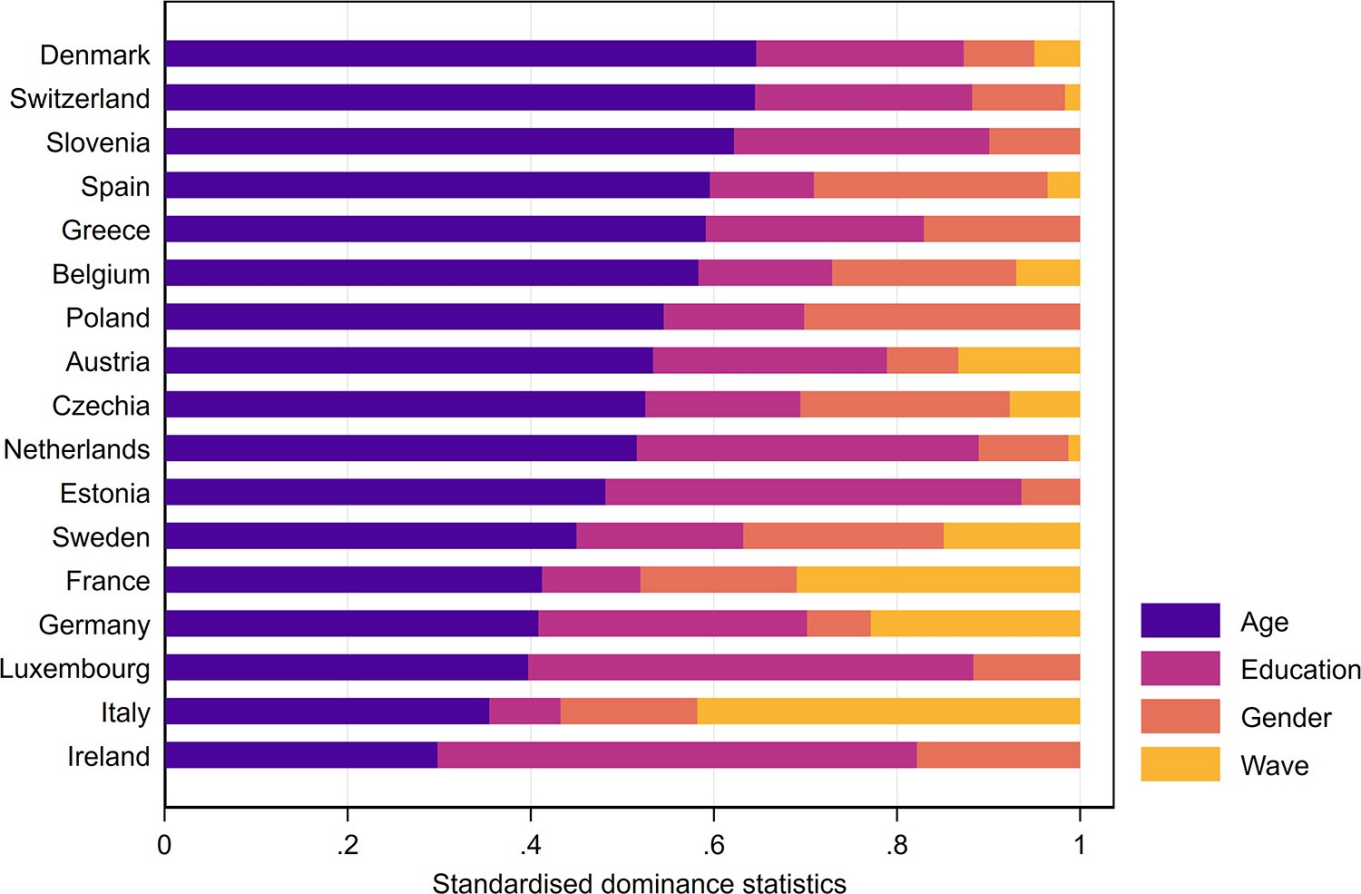
Decomposition of the overall bias in self-reported mobility.

Fig 3 shows the results of the relative importance analysis for each country individually. Because Estonia, Greece, Ireland, Luxembourg, Poland, and Slovenia only participated in one survey wave, the estimates of time effects for these countries are not provided. For the majority of the countries, age is the single most important characteristic explaining the bias of self-reported health. Depending on the country, either education or gender comes second. The contribution of time effects is negligible in most countries, except for France, Germany, and Italy. As discussed earlier, these time effects could be due to unobserved cohort effects, or because these countries changed their interview process between Wave 2 and Wave 5.

## Results on cognition

### Descriptive results

When asked about their memory, 29.4% of all survey participants report cognitive impairment (Table 1), yet when tested, only 16.1% recall three words or less. Overall, 71.8% of the participants show concordance between their reported and tested memories, but there is no clear difference between genders except for a slight tendency for men to overestimate and for women to underestimate their cognition. Concordance between mobility and cognition measures is highly related. According to Chi-squared tests, individuals that are prone to overestimate one dimension are also more likely overestimate the other; the same holds for underestimating and concordance.

Similar to mobility, there is a strong decline in concordance with age. While 80.6% of the 50–54 age group report their correct level of memory, only 63.9% of the 90–94 age group achieve concordance. Misreporting is even more pronounced at ages 80–84, in which 61.4% show divergence between tested and self-reported measures. Unlike mobility, it is not clear from the numbers whether the decrease in concordance with age is due to an increase in overestimating or underestimating. While the tendency to overestimate cognition increases steadily with age, under-estimating is highest at ages 75–79 (26.0%) and decreases thereafter.

There is a pronounced education gradient in the concordance between tested and self-reported cognition, where again Western and Northern countries have lower discrepancies. Switzerland has the highest rate of concordance (81.6%) and Estonia has the lowest (56.2%). However, the division is not as clear as for mobility, mainly because Sweden has a relatively low rate of concordance (71.0%), similar to that of Slovenia and Czechia.

### Regression analysis

Regression analyses also show concordance decreasing strongly with age (Table 3). Individuals aged 80–84 are three times as likely to overestimate their memory than the reference group of 60- to 64-year-olds (log odds 1.095). The oldest individuals, aged 90–94, are 3.7 times as likely to overestimate their cognitive ability (log odds 1.297). Similar to mobility, the probability to underestimate memory increases up to ages 75–79 (log odds 0.386), but slightly decreases again for the oldest individuals. Based on the country specific samples, S2 Fig provides the values of concordance by country and age. Contrary to mobility, the strong age gradient in concordance does not change once employment is controlled for (Table K in S1 Appendix).

**Table 3:** Multinomial logistic estimation for concordance between cognition measures.

	Overestimating	SE	Underestimating	SE
**Country (Ref: Slovenia)**				
Austria	-0.613***	0.066	-0.386***	0.053
Belgium	-0.392***	0.062	0.090	0.049
Czechia	-0.854***	0.066	0.251***	0.047
Denmark	-0.654***	0.076	-0.264***	0.058
Estonia	-0.690***	0.067	1.075***	0.045
France	-0.339***	0.061	0.332***	0.048
Germany	-0.473***	0.071	0.029	0.052
Hungary	-0.287***	0.086	0.495***	0.059
Italy	-0.325***	0.062	0.036	0.051
Luxembourg	-0.124	0.100	-0.429***	0.087
Netherlands	-0.622***	0.069	-0.499***	0.058
Poland	-0.072	0.098	0.201**	0.077
Portugal	-0.133	0.093	0.583***	0.068
Spain	-0.165**	0.059	0.058	0.049
Sweden	-0.686***	0.073	0.235***	0.051
Switzerland	-0.822***	0.076	-0.365***	0.058
**Age (Ref: 60–64)**				
50–54	-0.258***	0.056	-0.247***	0.032
55–59	-0.196***	0.049	-0.113***	0.027
65–69	0.162***	0.045	0.111***	0.026
70–74	0.526***	0.044	0.321***	0.028
75–79	0.885***	0.045	0.386***	0.030
80–84	1.095***	0.047	0.288***	0.035
85–89	1.182***	0.056	0.032	0.048
90–94	1.297***	0.085	-0.099	0.089
**Women**	-0.290***	0.025	0.091***	0.017
**Education (Ref: Medium)**				
Low	0.644***	0.031	0.240***	0.020
High	-0.445***	0.043	-0.308***	0.024
**Wave 5**	-0.127***	0.024	0.116***	0.015
Constant	-2.202***	0.059	-1.653***	0.046
N	113,812	Pseudo R^2^		0.055

Note: The dependent variable is a three-category variable that indicates if an individual achieved concordance (reference category), overestimated or underestimated his or her health. Coefficients are given in log odds, standard errors are clustered at the individual level,

*p<0.05,

**p<0.01,

***p<0.001

The effect of education on concordance is even stronger for cognition than it is for mobility. Less-educated participants are 1.9 times more likely to overestimate their memory (log odds 0.644) and 1.3 times more likely to underestimate their memory (log odds 0.240). Tertiary education is associated with a lower probability to both overestimate (log odds -0.445) and underestimate cognition (log odds -0.308). These results remain robust even after controlling for employment (Table K in S1 Appendix).

Contrary to mobility, women are less likely to overestimate their memory than men (log odds -0.290). However, females are slightly more likely to underestimate their cognition in the pooled model. In the country-specific estimations, this finding holds for Belgium, Estonia, France, Italy, Portugal, and Spain. However, in Austria, Denmark, and The Netherlands, women are less likely to underestimate their memory. The gender differences increase when memory impairment is based on delayed word recall, which indicates that women and men either interpret the subjective memory question differently, or relationship between immediate and delayed word recall differs between genders (Table E in S1 Appendix).

**Fig 4:**
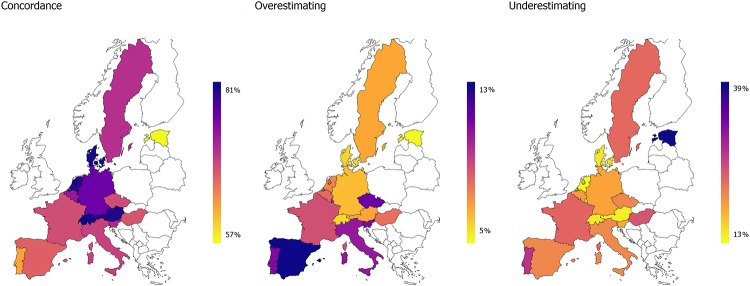
Concordance between tested and self-reported cognition by country (predicted shares).

Concordance between tested and self-reported cognition differs among the countries observed. Again, Southern European and CEE countries have lower rates of concordance than Western and Northern European countries (Fig 4). Two exceptions are Czech Republic, which achieves a relatively high rate of concordance, and Sweden, which achieves a medium level of concordance. As with mobility, the tendency to overestimate cognitive ability is much greater in Southern and CEE countries.

Interestingly, participants of Wave 5 are less likely to overestimate and instead more likely to underestimate. This finding does not change when additionally controlling for a potential learning effect (Table S in S1 Appendix). As with mobility, this could indicate a cohort and/or time effect or differences in the interview procedure over time, both of which the available data cannot account for. Finally, all results are robust to changes in the threshold of cognitive impairment (Tables C and D in S1 Appendix), to differences in the sample composition (Tables G and I in S1 Appendix) as well as to different model specifications (Tables K, M, O and Q in S1 Appendix).

### Relative importance analysis

The bias in self-reported cognition is mainly due to differences in reporting behaviour by country, which explain 44.9 per cent in the pooled model. Differences by age group contribute 29.7 per cent to the explained variation. Education is much more relevant in explaining the reporting bias in self-reported cognition (22.7 per cent) than it is for measures of mobility. Variations in reporting behaviour by gender (2.1 per cent) and survey wave (0.6 per cent) are even less important for self-reported memory than they are for self-reported mobility. This finding holds also when estimates are based on Wave 5 only (Tables H and I as well as Figs A and B in S1 Appendix).

**Fig 5:**
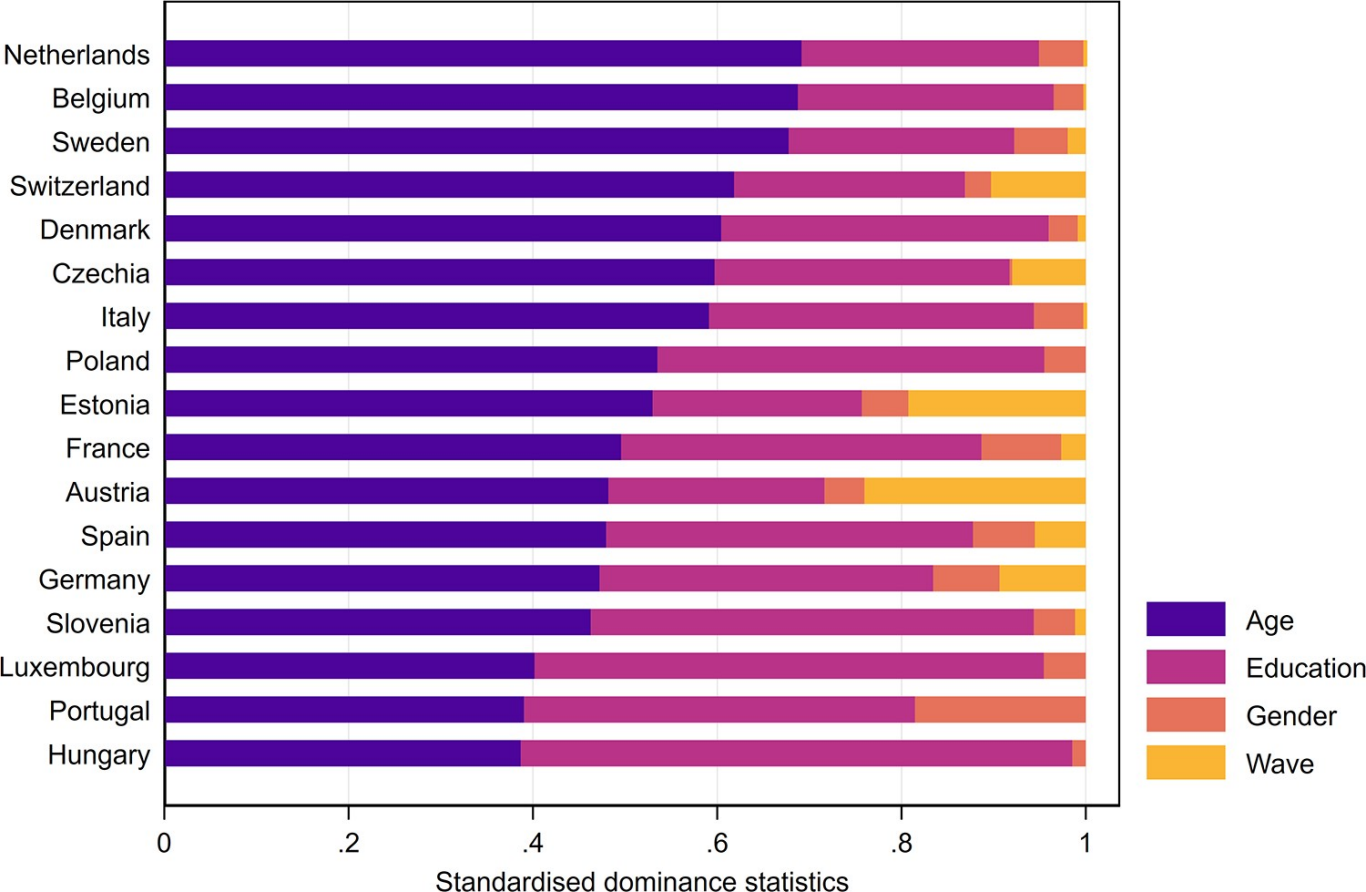
Decomposition of the overall bias in self-reported cognition.

Fig 5 shows country specific decompositions of the fit statistic. Age is still very relevant for explaining the reporting bias in cognition measures, yet education is just as important in some countries. On the contrary, gender and wave are neglectable when it comes to explaining the reporting bias. Two exceptions are Estonia and Austria, where the survey wave seems to contribute to the explained variance. Similar to the results on mobility, these exceptions could either be due to cohort effects, or because interviews were conducted differently in Wave 4 and Wave5.

## Discussion

In this study on older Europeans, we investigate the discrepancy between tested and self-reported health measures and explore which demographic characteristics are most important in explaining health misreporting. In particular, we focus on the demographic characteristics most frequently used for health comparisons, namely country of residence, gender, age and educational attainment. Furthermore, we investigate subordinate channels that might explain or mediate the effect of demographic characteristics on reporting behaviour, particularly employment status, parenthood and marital status. Conducting a relative importance analysis, we find that differences in reporting style between countries and age groups explain most of the bias in self-reported health. These findings suggest that comparisons of health between countries and age groups based on subjective data have to be treated particularly careful. In addition, for self-reported cognition specifically, misreporting varies substantially between educational groups. Parts of the strong age and education effects on reporting style can be explained by differences in employment by age and education. Parenthood and being married, however, add little to the bias. Sensitivity analyses show that the results are robust to changes in the definition of physical and cognitive impairment, sample composition and model specifications (S1 Appendix).

Concordance as well as the tendency to overestimate and underestimate health vary strongly across Europe. Results from the relative importance analyses show that 35% of the reporting bias in mobility and 45% of the bias in memory are due to differences in reporting behaviour between countries. Overall, Northern and Western European countries have fewer discrepancies than CEE or Southern European countries. Southern Europeans seem particularly prone to overestimating their health, which is contrary to the results of [14], who finds that Scandinavians overrate their health the most. Previous studies also identified country differences in reporting style for European countries [14,46,47], low- and middle-income countries [4], as well as within countries and across subpopulations [5]. It was shown that self-reports are influenced by culture-specific reporting behaviour, compositional differences between countries and differences in the perception of how restricting poor health is [11]. In addition, the strong country effects could also be due to different health care policies. For instance, the proportion of elderly persons in residential care varies across Europe, thus frail persons might be sampled differently across countries. If frailty affects response behaviour, different shares of frail individuals in the country samples could explain differences in aggregated concordance. We controlled for this possibility by excluding all frail individuals from the analysis, yet the results remained robust (Tables F and G in S1 Appendix). Speculatively, the between-country discrepancies could also be due to differences in regional development. For a subset of our country sample, early results on the relationship between a regional developmental index [48] and discrepancies in mobility suggest that countries with better living conditions show more concordance than their counterparts. However, further research with data on the whole lifecycle is needed to investigate the potential development effect properly.

In addition to the cultural bias in self-reported data, we find a strong decrease in concordance with age for both health dimensions. This result is in accordance with earlier research on several physical performance measures [6–8]. Further, previous research supports our finding that subjective health measures of older individuals are often upward biased [35]. One explanation could be that octogenarians and nonagenarians tend to compare their health status with peers suffering from worse health, which enables them to maintain a positive perception of their own health state [36]. This so called downward comparison makes older persons feel more satisfied with their lives, especially, when they are frail themselves [49]. Resilience strategies like these help individuals to flexibly adapt to changes of their physical and cognitive health while maintaining a positive self-image [50].

Overall, the age-related decline in concordance between performance based and perceived memory measures is robust to controlling for employment (Table K in S1 Appendix). However, concordance between mobility measures declines less steeply with age once the employment status is considered. This indicates that a part of the strong age effect is due to variation in the share of employed persons across age groups. The causal direction, however, remains unclear. It could either be that employed individuals are more aware of their physical ability, or that persons that are more aware of their own health status are more likely to be employed. Thus, future studies could fruitfully explore the interrelations between health perception, age and employment.

We also identify a clear education gradient in concordance for mobility and an even stronger effect for cognition. Less-educated individuals tend to misreport their mobility and memory more frequently, whereas the highly educated are less likely to misreport. Previous research does not provide conclusive results on this matter. Some studies report that higher education results in a more optimistic view on health [8], while others find the exact opposite [33,51,52] or no significant education effect at all [53,54]. Overall, our results on education can be interpreted as additional evidence for the phenomenon that higher educated individuals have higher health awareness and literacy [55,56]. For example, higher educated are more familiar with the risks of tobacco smoking [57], less likely to misjudge their weight [58] and, as shown in this study, also less likely to have a biased view on their physical and cognitive abilities. Since health literacy is an important determinant of health behaviour and consequently health itself [59–61], enhancing health literacy of low educated individuals could improve their health outcomes. It may also be hypothesised that the gender gap in the education of older Europeans contributes to differences in misreporting. On average, men at advanced age are higher educated than women within our investigated cohorts. What supports this hypothesis is our finding that less-educated women are particularly prone to underestimate their mobility (Table P in S1 Appendix). In addition, employment status at higher ages varies by gender and education with higher educated being more likely to work longer [62]. Our robustness analyses showed that the education gradient in concordance appears less pronounced for mobility once employment is accounted for, but interestingly does not change for cognition (Tables J and K in S1 Appendix). The educational differences in cognition only changed when delayed word recall is used and education is less important to explain the differences (Table O in S1 Appendix).

We also find differences in reporting behaviour between men and women, but they are less pronounced and explain very little of the overall reporting bias. In particular, women tend to underestimate their health more frequently in both health dimensions. One explanation for these gender differences might be the tendency of women to report limitations more frequently [37–39], while men tend to underreport their health status [63]. Recent research also showed that reporting morbidity was more legitimate in female-dominated work environments, indicating an association of gender norms with gender difference in reporting behaviour [39]. This might also be related to women looking for medical advice more often than men [64,65]. Interestingly, our findings on overestimating health vary by health dimension with women being less likely to overestimate their memory than men, but being more likely to overestimate their mobility. Moreover, difference between genders increases when delayed word recall is instead of immediate word recall, which indicates that women and men might interpret the subjective memory question differently. Our small and sometimes ambiguous gender effects are in line with the literature, which does not provide conclusive results either. While some studies comparing self-assessed and clinical data find clear evidence that women are more likely to overestimate their health [66], others identify women to be more likely to underestimate their health [67,68]. A recent study based on SHARE data found no clear gender-specific pattern in reporting behaviour [15].

In general, our results not only give guidance on how to carefully interpret self-reported health measures, but might also contribute to a reduction in adverse health outcomes due to mistaken self-assessments. For instance, overestimating lower body functioning might contribute to higher risks of fall-induced injuries [10]. Further, overestimating cognitive abilities might result in illusory self-awareness of everyday functioning [69]. In psychology, the consequences of wrong self-awareness of cognitive abilities are discussed as the Dunning-Kruger effect, which states that unable individuals are especially prone to overestimate their abilities [70,71]. If the tendency to overestimate ones physical and cognitive capacity has an adverse impact on health-related behaviour of older Europeans, then awareness should be created in particular among the oldest old, among men and among Southern Europeans.

A major contribution to the literature is that we are able to compare reporting behaviour of mobility and cognition simultaneously. The results show that concordance of the two health dimensions is highly related. Individuals that are prone to misreport one dimension are also more likely to misreport the other. This indicates that correlations between the two health dimensions are, to a certain degree, due to similarities in reporting behaviour. However, we also find differences in the reporting styles of subjective physical and cognitive health. For instance, concordance is slightly higher between mobility measures than between memory measures. Furthermore, the composition of the bias in self-reports differs between the two health dimensions. The cultural bias in subjective data, i.e. differences across countries, is more relevant for cognition than for mobility. Additionally, reporting heterogeneities between education groups result in larger biases in self-reported memory than in self-reported mobility. Gender, however, explains relatively little of the bias in both health dimensions.

Controlling for wave effects shows that participants in Wave 5 are less likely to overestimate their mobility as well as their cognition, even after controlling for potential learning effects. These findings indicate that cohort or time effects influence the reporting style, which is crucial since the analysis of mobility and memory are based on different waves. To ensure that the differences in reporting style of physical and cognitive health do not stem from differences in the sample composition, we conducted a robustness analysis for which we restricted our analysis to Wave 5, which is the only wave that provides relevant data for mobility and memory. Tables H and I in S1 Appendix show that the overall findings remain even after both health dimensions are analysed based on the same subsample.

The main limitations of this study are threefold. First, the population composition is likely to vary across countries. We conducted robustness analyses for different sample shares of frail individuals, but additional deviations in the sample composition could also influence the results. Second, the questionnaire is conducted in the national language, which could result in some bias when it comes to self-assessed health because the wording differs across languages. Third, it appears that some of the effects are influenced by time or cohort effects, however, disentangling these effects is not feasible with the data at hands.

In conclusion, self-reported measures of mobility and cognition have to be treated cautiously, in particular when comparing health across countries and age groups. In addition, the education gradient in concordance needs to be considered when analysing memory. Finally, men and women show different reporting behaviours, yet the impact of gender on the overall bias between tested and self-reported health is less pronounced than that of other demographic characteristics.

## Supporting information

S1 Appendix Robustness analyses. (PDF)

S1 Fig Predicted values of concordance between tested and self-reported mobility by country and age. (TIF)

S2 Fig Predicted values of concordance between tested and self-reported cognition by country and age. (TIF)
