# Supplementary material for: Reporting biases in self-assessed physical and cognitive health status of older Europeans
Source: PLoS One. 2019 Oct 8;14(10):e0223526. doi: 10.1371/journal.pone.0223526 (PMC6783110; doi:10.1371/journal.pone.0223526)
Supplement: S1 Appendix — (PDF) [file pone.0223526.s001.pdf]

S1 Appendix. Robustness analyses

We conduct a range of robustness analyses to investigate whether the results presented in the main document are sensitive to changes in the definition of physical and cognitive impairment, sample composition and model specifications. Furthermore, we explore additional channels that may help to explain the effects of demographic characteristics on concordance, overestimating and underestimating.

Additional definitions of physical impairment

As a robustness analysis, we apply a stricter scenario where individuals are considered physically impaired when they have to use their arms to stand up from the chair, which is considered unimpaired in the main analysis. All trends described in the main text hold (Table A). Most of the average values are very similar to those when individuals are allowed to use their arms. However, there is slightly less concordance and a small increase in overestimating when individuals are not allowed to use their arms. This shift is plausible, since the question on mobility does not ask whether or not individuals use their arms when standing up from a chair. Respondents simply might not interpret having to use their arms as an impairment.

An additional specification of impairment is also applied, for which individuals who think it is unsafe to try the chair stand test are excluded from the analysis rather than considering them as impaired (Table A). The reduced sample includes 73,912 observations instead of 88,087. As expected, this specification alters the results. Concordance increases in each subgroup, mainly because overestimating drops to only 0.9%. This indicates that individuals that are unable to stand up from a chair avoid the test in the first place rather than failing the test. Individuals who report having no problem getting up from a chair might prefer not to be tested if they expect to perform badly at the test. Even though the level of overestimating is much lower with the new specification, most observed trends still hold. Concordance is still higher for men and highly educated individuals and decreases with age. Yet, the results by country vary from those in the first specifications. All Central and Eastern European (CEE) countries are still in the bottom half of concordance, but Southern European countries have higher relative rates of concordance in the new specification since large numbers of overestimating respondents are dropped in that specification. Still, most Southern and CEE European countries as well as Ireland have above-average rates of overestimation. While Northern European countries still have above-average concordance, Western European countries have a scattered distribution of results using this new specification.

Table B displays results of applying Models 1 and 2 on the reduced sample (i.e. where everyone refusing to do the chair stand test is dropped). We find that Southern European countries have much higher concordance rates for mobility measures. Furthermore, Austria, Germany, and Luxembourg have relatively low concordance, as their tendency to underestimate mobility is relatively more important. Concordance still decreases with age, mainly due to an increase in underestimating opposed to an increase in overestimating. In summary, overestimating may mainly be due to not taking part in the test, which is especially relevant

for Southern European and CEE countries. Once these observations are dropped, underestimating is more prevalent, especially in Western European countries. Similar to the descriptive evidence, these results indicate self-selection of individuals in performing the test.

Additional definitions of cognitive impairment

Table C provides summary statistics for an additional specification of cognitive impairment. In our main analysis, individuals are considered to be cognitively impaired if they recall three words or less in the memory test. For this sensitivity analysis, a more lenient threshold is applied in which participants are considered to be impaired when they recall two words or less. Applying this specification results in a much lower proportion of impaired individuals (7.6% versus 16.1% using the original specification). While the overall rate of concordance hardly changes, the tendency to overestimate is much lower and the tendency to underestimate is much higher with the new specification. This is to be expected as the new specification considers fewer individuals to be impaired.

Although the overall levels of overestimating and underestimating change with the new specification, the trends observed in the main analysis hold. Men are still more likely to achieve concordance than women. While men tend to overestimate their cognition, women tend to underestimate theirs. The result still shows a clear decrease in concordance with age and both overestimating and underestimating show the same patterns with age as with the original specification of impairment. We still observe a strong education gradient in concordance and the country ranking is almost identical to that of the original specification. Switzerland has still the highest rate of concordance (83.2%), while Estonia has the lowest (53.1%).

Table D displays the regression results for Models 1 and 2 when using the new specification of cognitive impairment. The magnitude of the coefficients changes, yet the findings remain the same as within the main analysis. The pattern of age effects and between countries are almost identical to the main findings. The only difference is that the level of overestimating is lower and the level of underestimating is higher with the new specification. In conclusion, the threshold of impairment impacts the level of overestimating and underestimating, but not the overall trends in concordance between tested and self-reported cognition.

In our main analysis, objective cognition was based on immediate word recall. However, the self-assessment of memory might also refer to delayed word recall. Thus, we also provide an additional analysis of objective cognitive impairment based on delayed word recall. During the interview, survey participants are first asked to repeat a list of ten words, which is the basis for the immediate word recall measure. Following that, the participants perform some additional tests, for example on numeracy. After these additional tests, which take approximately 5 minutes, the interviewer asks "A little while ago, I read you a list of words and you repeated the ones you could remember. Please tell me any of the words that you can remember now?", which is the basis for a delayed word recall measure. While survey participants recall on average 5.2 words immediately, they only recall 3.9 words in the delayed test. As a consequence,

concordance is lower when objective cognition is based on delayed word recall, because, by default, more individuals overestimate their cognition when the new definition is applied.

Table E presents regression results for when objective cognition is based on delayed word recall. While the trend in age is similar to that of immediate word recall, the decrease in concordance with age appears less steep. Furthermore, differences between educational attainment groups are smaller when the new specification is applied. On the contrary, the difference between the genders increases. In line with these findings, the results based on the relative importance analysis show that age and education appear slightly less important in explaining the variance in response behaviour, whereas gender appears more relevant. The main conclusions and the relative ranking of determinants remain. Specifically, in the model with immediate (delayed) word recall, country differences contribute 45% (45%) to the explained variance, age differences 30% (24%), educational differences 23% (21%), gender 2% (8%) and time effects 1% (2%).

Additional sample compositions

We also analyse whether the results are sensitive to different sample compositions. For example, frail individuals might be more likely to live in institutions in some countries than in other countries and consequently are not always included in our target population of non-institutionalised population. This could be relevant for the results since the survey respondent's overall level of health might affect concordance, especially when they suffer from very poor health. Thus, we exclude frail individuals from the sample and analyse if they influence the outcomes. To measure frailty, we rely on a well-established indicator introduced by [1], for which individuals are considered frail if they show three or more of the following components: exhaustion, weakness, slowness, shrinking and low activity levels. We follow exactly the operationalisation by [2], who adapted the indicator for SHARE data. According to the frailty measure, 8% of the survey participants are considered frail in our mobility sample (Waves 2 and 5), and 9% in our cognition sample (Waves 4 and 5). Consequently, 6,335 observations are dropped for the robustness analysis of mobility, and 9,996 observations for cognition.

The results for mobility are presented in Table F. Country coefficients change marginally in magnitude when frail individuals are excluded, while all other coefficients remain almost identical. Similarly, results based on relative importance analysis hardly change when frail survey participants are dropped. In the model with (without) frail individuals, country differences contribute 35% (39%) to the explained variance, age differences contribute 29% (32%), education differences contribute 17% (15%), gender differences contribute 11% (11%) and time effects contribute 5% (6%). Thus, the only difference is that age and education contribute marginally less to the explained variance in concordance, which appears plausible since frailty is highly correlated with age and education. Consequently, all other determinants explain relatively more of the variation once frailty is accounted for. The results for cognition hardly change when frail individuals are dropped from the sample (Table G). Country coefficients change slightly in magnitude, but not in sign. All other coefficients are virtually identical to those of the main regression analysis. Similarly,

results based on relative importance analysis remain unaffected. In summary, the results appear robust to different compositions of frail individuals and their reporting behaviour.

In the main analysis, we describe differences in reporting behaviour between physical and cognitive impairment. Physical impairment is taken from Wave 2 and Wave 5, cognitive impairment from Wave 4 and Wave 5. Since the results for the two health dimensions are not based on the same sample, these differences could stem from differences in the sample rather than differences in reporting behaviour. Thus, we run additional analyses based on Wave 5 only, in which information on concordance of physical as well as cognitive health care measures is provided, i.e. we can estimate the relationship between demographic characteristics and the probability to overestimate or underestimate physical and cognitive health based on the exact same group of individuals. The regression results are provided in Table H and Table I. Since wave dummies are not needed for this specification, they are excluded from the model. Although some of the coefficients slightly change in magnitude and significance, the main results appear robust. Results from the relative importance analysis cannot be directly compared with the main model, since the wave dummy is now missing. In Wave 5, the explained variation in concordance of mobility measures can be decomposed as follows: country differences 29%, age differences 43%, educational differences 19%, gender differences 10%; thus, the main difference to the estimations based on both waves is that age appears more relevant now than when both waves are combined. The variation in concordance of cognition measures can be decomposed as follows: country differences 50%, age differences 27%, educational differences 21% and gender 1%. Thus, the results are very similar to the main computations. Results for each country individually can be found in Figs A and B.

Additional model specifications

In addition to demographic characteristics, other factors might have an impact on concordance and/or further explain the effect of demographic characteristics on reporting behaviour. In particular, we analyse whether the results change after we account for employment status, marital status and whether a person has children (Tables J-O). Furthermore, Tables P to S provide regression results including learning effects and an interaction term between gender and education.

Whether an individual works or not is likely to influence health perception. First, persons working regularly might be more aware of their mobility impairments. Further, during their working tasks they might face limitations of their memory abilities, which might be particularly relevant for individuals working in analytical jobs. Since age is highly correlated with an individual's employment status, parts of the strong effect of age on concordance might be explained by younger survey participants that are still in employment. Furthermore, employment might be an important mediator for the effect of educational attainment on concordance between measures of cognitive health, since highly educated individuals are more likely to work in jobs that demand strong cognitive skills. To test the employment channel, we add a dummy variable

to the models that indicates if an individual is employed, as opposed to retired, unemployed, permanently sick or a homemaker.

In the mobility sample, 27% of the survey participants are employed and in the cognition sample, it is 26%. In both samples, employment has a strong negative correlation with age and a strong positive correlation with educational attainment. Furthermore, summary statistics show that employed individuals are more likely to achieve concordance. Tables J and K present regression results for mobility and cognition respectively. As expected, employed individuals are less likely to overestimate or underestimate their physical and cognitive health. Furthermore, the age gradient in concordance appears less pronounced. In addition, the education gradient in concordance appears less pronounced for mobility once employment is accounted for but does not change for cognition.

In addition to employment, having children or being in a relationship might influence health perception. For example, if family members comment on the survey participant's health status or if the health of other family members serves as a reference point. Thus, we provide results for two more models, in which we control for whether the survey participant has children (Tables L and M) and for whether the survey participant is married or in a registered partnership (Tables N and O). The coefficients for children and marriage either have the expected sign or are insignificant. What is more relevant for the work at hands, however, is that the inclusion of these variables has almost no impact on all other coefficients.

Relative importance analysis confirms that the employment channel explains part of the strong age effect, at least for reporting behaviour related to mobility. When employment status, marital status and a dummy for children are added to the model for mobility, country differences still contribute 32% percent to the explained variation, but age differences drop to 20%, probably, because differences in employment status explain 17%. Likely, for the same reason, the contribution of educational differences slightly decreases to 13%. Gender remains at 9% and wave at 4%. Being married (3%) and having children (1%) explains only little of the variation. Similar results are found for cognition, although employment seems relatively less important in explaining concordance. Country differences contribute 44% to the explained variation, age differences 22%, differences in employment status 11%, educational differences 20%, gender 2% and wave less than one per cent. Again, the contribution of having children and being married is negligible.

Including additional mediators in the model identified potential pathways, but more detailed analyses are required to draw concrete conclusions. For instance, the effect of labour market participation should be investigated more thoroughly considering factors such as the number of working hours, part-time retirement and type of occupation; however, this goes beyond the scope of this study.

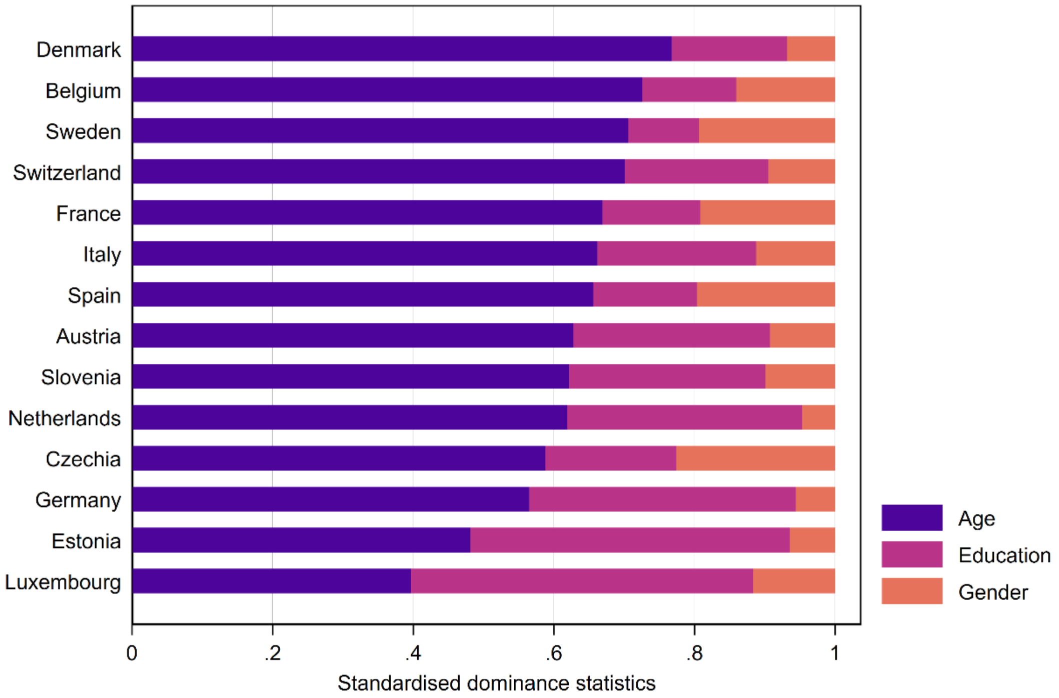

Fig A. Decomposition of the overall bias in self-reported mobility (based on Wave 5 only)

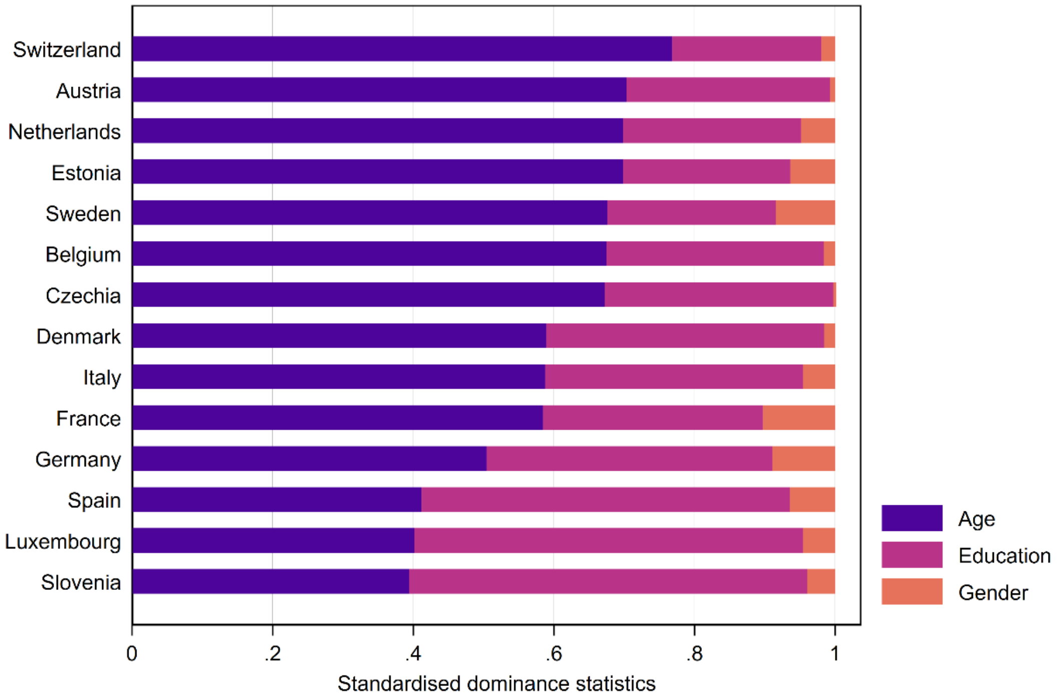

Fig B. Decomposition of the overall bias in self-reported cognition (based on Wave 5 only)

Table A. Summary statistics showing different specifications of impaired mobility

	Chair stand without using arms						Chair stand without participants that felt unsafe					
	Impairment		Concordance				Impairment		Concordance			
	S	T	S=T	S>T	S<T		S	T	S=T	S > T	S < T	
	%	%	%	%	%	N	%	%	%	%	%	N
**Total**	19.2	18.0	80.0	10.0	9.9	88,087	19.2	1.3	86.9	0.9	12.1	73,912
**Gender**												
Men	14.9	16.0	82.5	9.8	7.7	39,417	14.9	1.2	89.8	1.0	9.2	33,832
Women	22.7	19.7	78.1	10.2	11.7	48,670	22.7	1.4	84.5	0.9	14.6	40,080
**Age**	10.3	10.5	85.2	7.5	7.3	11,229	10.3	1.1	90.9	1.0	8.1	10,219
50–54	12.7	12.2	83.7	8.0	8.4	16,196	12.7	1.3	89.4	1.0	9.5	14,501
55–59	14.9	13.1	82.0	8.1	9.9	16,836	14.9	1.0	87.9	0.8	11.3	14,886
60–64	16.6	15.5	80.0	9.5	10.5	15,721	16.6	1.1	86.6	0.9	12.6	13,569
65–69	20.7	20.6	77.5	11.3	11.2	12,906	20.7	1.5	84.9	1.0	14.1	10,553
70–74	26.9	26.1	75.4	12.4	12.2	7,347	26.9	1.2	82.8	0.7	16.5	5,579
75–79	34.4	38.2	71.0	16.8	12.2	4,664	34.4	1.9	79.1	1.2	19.7	3,012
80–84	42.6	52.1	68.3	21.1	10.6	2,438	42.6	4.4	76.1	2.2	21.7	1,281
85–89	46.9	62.2	65.2	25.9	8.9	750	46.9	4.2	73.4	3.2	23.4	312
90–94	10.3	10.5	85.2	7.5	7.3	11,229	10.3	1.1	90.9	1.0	8.1	10,219
**Education**												
Low	24.7	25.0	75.9	13.1	11.0	35,808	24.7	1.8	84.1	1.2	14.7	27,858
Medium	16.9	15.0	81.2	8.8	10.1	31,953	16.9	1.1	87.3	0.8	11.9	27,644
High	11.8	10.0	86.0	6.3	7.6	19,058	11.8	0.7	90.9	0.6	8.5	17,374
**Country**												
Austria	20.8	18.3	79.9	9.2	10.8	5,032	20.8	1.2	86.0	0.8	13.2	4,182
Belgium	19.5	14.6	80.7	7.6	11.7	7,932	19.5	0.5	85.9	0.4	13.7	6,845
Czechia	23.2	22.7	77.8	11.4	10.7	7,651	23.2	1.3	84.9	1.0	14.1	6,102
Denmark	12.7	7.7	87.6	4.3	8.1	6,014	12.7	0.3	91.1	0.2	8.7	5,578
Estonia	29.1	26.9	76.5	10.7	12.8	5,454	29.1	1.4	81.6	1.0	17.5	4,079
France	16.3	17.6	79.8	11.3	8.9	6,566	16.3	2.3	87.8	1.6	10.6	5,563
Germany	19.6	14.4	80.1	7.9	12.0	7,700	19.6	1.1	85.2	0.8	13.9	6,712
Greece	18.1	21.5	77.5	15.5	7.0	2,601	18.1	0.8	89.8	0.7	9.5	2,133
Ireland	18.0	20.6	77.8	14.1	8.1	792	18.0	2.8	88.0	2.2	9.8	651
Italy	19.4	25.8	75.6	16.0	8.4	6,919	19.4	2.5	86.8	1.7	11.5	5,383
Luxembourg	21.2	16.5	78.5	8.6	12.9	1,561	21.2	0.7	84.2	0.5	15.3	1,318
Netherlands	14.7	10.4	85.6	5.4	9.0	6,258	14.7	0.6	89.7	0.3	10.0	5,663
Poland	29.5	29.7	70.5	17.1	12.3	1,969	29.5	3.7	79.9	3.0	17.2	1,445
Slovenia	20.9	20.1	78.0	10.8	11.2	2,873	20.9	0.5	85.3	0.4	14.3	2,325
Spain	21.8	27.0	76.7	15.3	7.9	8,011	21.8	2.4	87.1	2.0	10.9	6,207
Sweden	15.4	11.3	83.6	6.7	9.6	6,611	15.4	0.7	88.6	0.5	10.9	5,932
Switzerland	11.2	9.9	85.3	7.0	7.7	4,143	11.2	1.0	90.6	0.8	8.6	3,794
**Wave**												
Wave 2	18.6	17.7	79.4	11.7	8.9	26,973	18.6	1.6	87.9	1.2	10.9	22,867
Wave 5	19.5	18.2	80.3	9.3	10.3	61,114	19.5	1.1	86.5	0.8	12.7	51,045

Note: S refers to self-reported impairment and T refers to tested impairment. S=T denotes concordance, S>T denotes overestimating, and S<T denotes underestimating. N = 100%

Table B. Multinomial logistic estimation for concordance between mobility measures (excl. participants that felt unsafe)

	Overestimating	SE	Underestimating	SE
**Country (Ref: Slovenia)**				
Austria	0.745*	0.375	-0.052	0.077
Belgium	-0.141	0.391	0.046	0.072
Czechia	0.840*	0.363	-0.022	0.072
Denmark	-0.790	0.452	-0.379***	0.080
Estonia	0.958**	0.370	0.222**	0.073
France	1.210***	0.354	-0.333***	0.076
Germany	0.743*	0.360	0.141*	0.071
Greece	0.131	0.440	-0.328***	0.099
Ireland	1.424**	0.441	-0.155	0.149
Italy	1.254***	0.356	-0.296***	0.076
Luxembourg	0.142	0.531	0.139	0.099
Netherlands	-0.366	0.413	-0.364***	0.077
Poland	1.785***	0.379	0.413***	0.097
Spain	1.410***	0.356	-0.396***	0.075
Sweden	0.10	0.385	-0.290***	0.075
Switzerland	0.602	0.380	-0.543***	0.085
**Age (Ref: 60–64)**				
50–54	0.177	0.141	-0.383***	0.045
55–59	0.298*	0.126	-0.190***	0.038
65–69	0.121	0.134	0.116**	0.037
70–74	0.230	0.138	0.220***	0.039
75–79	-0.008	0.194	0.382***	0.046
80–84	0.668***	0.196	0.604***	0.055
85–89	1.244***	0.225	0.728***	0.075
90–94	1.733***	0.344	0.853***	0.145
**Women**	0.020	0.078	0.516***	0.025
**Education (Ref: Medium)**				
Low	0.234*	0.096	0.229***	0.029
High	-0.141	0.119	-0.325***	0.035
**Wave 5**	-0.351***	0.093	0.018	0.029
Constant	-5.336***	0.355	-2.234***	0.073
N	72,876	Pseudo R^2		0.036

Note: The dependent variable is a three-category variable that indicates if an individual achieved concordance (reference category), overestimated or underestimated his or her health. Coefficients are given in log odds, standard errors are clustered at the individual level, *p<0.05, **p<0.01, ***p<0.001

Table C. Summary statistics showing a different specification of impaired cognition

**Cognition, impaired if able to recall two words or less**						
	**Impairment**		**Concordance**			
	S	T	S=T	S>T	S<T	
	%	%	%	%	%	N
**Total**	29.4	7.6	72.4	3.0	24.7	115,785
**Gender**						
Men	28.1	7.8	73.2	3.3	23.5	51,013
Women	30.4	7.4	71.7	2.7	25.6	64,772
**Age**						
50–54	17.6	2.7	81.6	1.7	16.7	13,244
55–59	20.5	2.8	79.0	1.6	19.4	19,461
60–64	22.9	3.4	76.8	1.8	21.4	21,098
65–69	26.5	4.3	73.7	2.1	24.2	19,447
70–74	33.8	7.0	67.3	3.0	29.7	16,180
75–79	42.0	12.7	61.8	4.6	33.7	12,350
80–84	48.5	21.3	60.3	6.4	33.3	8,525
85–89	52.3	30.6	62.2	8.3	29.5	4,283
90–94	53.2	37.7	64.4	10.5	25.1	1,197
**Education**						
Low	39.7	13.4	64.5	4.7	30.8	46,113
Medium	24.8	4.0	75.5	1.9	22.6	43,362
High	17.7	2.7	81.8	1.6	16.6	24,337
**Country**						
Austria	17.8	5.7	81.9	2.9	15.2	9,028
Belgium	24.4	6.5	75.1	3.5	21.3	10,511
Czechia	30.0	4.9	72.1	1.5	26.4	10,609
Denmark	17.3	3.8	82.9	1.8	15.3	6,171
Estonia	51.4	8.2	53.1	1.8	45.0	11,792
France	31.9	8.4	69.3	3.6	27.2	9,796
Germany	22.4	4.8	77.7	2.4	19.8	7,099
Hungary	34.2	7.9	67.4	3.2	29.4	2,938
Italy	32.9	11.1	70.7	3.9	25.3	7,895
Luxembourg	18.5	7.4	79.6	4.8	15.6	1,543
Netherlands	15.7	4.4	83.1	2.8	14.1	6,770
Poland	32.8	12.1	70.0	4.5	25.5	1,678
Portugal	45.4	13.9	59.1	4.6	36.3	1,899
Slovenia	26.9	8.7	74.2	3.9	21.9	5,511
Spain	41.1	17.4	65.8	5.1	29.0	9,628
Sweden	29.3	4.9	71.8	2.1	26.1	6,346
Switzerland	16.5	3.0	83.2	1.8	15.1	6,571
**Wave**						
Wave 4	29.4	7.9	72.2	3.1	24.7	55,172
Wave 5	29.4	7.2	72.6	2.8	24.6	60,613

Note: S refers to self-reported impairment and T refers to tested impairment. S=T denotes concordance, S>T denotes overestimating, and S<T denotes underestimating. N = 100%

Table D. Multinomial logistic estimation for concordance between cognition measures (impaired if able to recall two words or less)

	Overestimating	SE	Underestimating	SE
**Country (Ref: Slovenia)**				
Austria	-0.337***	0.098	-0.419***	0.049
Belgium	-0.150	0.094	0.004	0.046
Czechia	-0.983***	0.110	0.169***	0.043
Denmark	-0.676***	0.122	-0.326***	0.054
Estonia	-0.400***	0.101	1.079***	0.042
France	-0.192*	0.093	0.267***	0.045
Germany	-0.267*	0.107	-0.048	0.049
Hungary	-0.047	0.130	0.496***	0.055
Italy	-0.249**	0.096	0.000	0.046
Luxembourg	0.173	0.142	-0.445***	0.080
Netherlands	-0.560***	0.107	-0.597***	0.055
Poland	0.088	0.147	0.209**	0.071
Portugal	0.130	0.138	0.662***	0.063
Spain	-0.037	0.090	0.169***	0.045
Sweden	-0.747***	0.118	0.142**	0.048
Switzerland	-0.832***	0.120	-0.448***	0.055
**Age (Ref: 60–64)**				
50–54	-0.065	0.087	-0.247***	0.031
55–59	-0.146	0.079	-0.114***	0.027
65–69	0.151*	0.074	0.158***	0.025
70–74	0.569***	0.071	0.411***	0.026
75–79	0.985***	0.070	0.562***	0.028
80–84	1.307***	0.071	0.554***	0.032
85–89	1.502***	0.080	0.399***	0.042
90–94	1.703***	0.113	0.211**	0.079
**Women**	-0.295***	0.037	0.052**	0.016
**Education (Ref: Medium)**				
Low	0.747***	0.047	0.361***	0.019
High	-0.273***	0.065	-0.359***	0.024
**Wave 5**	-0.107**	0.037	0.099***	0.014
Constant	-3.463***	0.092	-1.546***	0.042
N	113,812	Pseudo R-squared		0.063

Note: The dependent variable is a three-category variable that indicates if an individual achieved concordance (reference category), overestimated or underestimated his or her health. Coefficients are given in log odds, standard errors are clustered at the individual level, *p<0.05, **p<0.01, ***p<0.001

Table E. Multinomial logistic estimation for concordance between cognition measures (delayed word recall)

	**Overestimating**	**SE**	**Underestimating**	**SE**
**Country (Ref: Slovenia)**				
Austria	-0.653***	0.042	-0.343***	0.070
Belgium	-0.453***	0.040	0.304***	0.063
Czechia	-0.463***	0.039	0.279***	0.062
Denmark	-0.731***	0.047	-0.117	0.073
Estonia	-0.890***	0.042	0.974***	0.058
France	-0.583***	0.041	0.532***	0.062
Germany	-0.491***	0.043	0.059	0.068
Hungary	-0.510***	0.055	0.332***	0.080
Italy	-0.342***	0.042	0.061	0.068
Luxembourg	-0.465***	0.067	-0.235*	0.109
Netherlands	-0.562***	0.044	-0.226**	0.074
Poland	-0.151*	0.065	-0.172	0.118
Portugal	-0.532***	0.064	0.571***	0.088
Spain	-0.363***	0.041	-0.109	0.068
Sweden	-0.755***	0.046	0.425***	0.066
Switzerland	-0.863***	0.047	-0.146*	0.072
**Age (Ref: 60–64)**				
50–54	-0.257***	0.030	-0.159***	0.038
55–59	-0.151***	0.026	-0.022	0.033
65–69	0.176***	0.025	0.076*	0.032
70–74	0.339***	0.026	0.159***	0.034
75–79	0.414***	0.028	0.018	0.039
80–84	0.484***	0.032	-0.223***	0.048
85–89	0.472***	0.040	-0.621***	0.070
90–94	0.609***	0.069	-0.847***	0.152
**Women**	-0.307***	0.015	0.178***	0.021
**Education (Ref: Medium)**				
Low	0.259***	0.018	0.007	0.025
High	-0.429***	0.023	-0.205***	0.028
**Wave 5**	-0.125***	0.014	0.134***	0.019
Constant	-0.364***	-0.037	-2.058***	-0.060
N	113,721	Pseudo R-squared		0.036

Note: The dependent variable is a three-category variable that indicates if an individual achieved concordance (reference category), overestimated or underestimated his or her health. Coefficients are given in log odds, standard errors are clustered at the individual level, *p<0.05, **p<0.01, ***p<0.001

Table F. Multinomial logistic estimation for concordance between mobility measures (frail individuals are excluded from the sample)

	**Overestimating**	**SE**	**Underestimating**	**SE**
**Country (Ref: Slovenia)**				
Austria	-0.266**	0.086	-0.041	0.079
Belgium	-0.542***	0.084	0.055	0.075
Czechia	-0.074	0.079	-0.045	0.074
Denmark	-1.112***	0.100	-0.341***	0.083
Estonia	0.004	0.082	0.102	0.076
France	-0.136	0.080	-0.340***	0.081
Germany	-0.341***	0.081	0.166*	0.074
Greece	-0.013	0.094	-0.356***	0.103
Ireland	0.152	0.130	-0.237	0.158
Italy	0.228**	0.077	-0.391***	0.081
Luxembourg	-0.242*	0.121	0.113	0.103
Netherlands	-0.974***	0.093	-0.305***	0.080
Poland	0.352***	0.098	0.304**	0.101
Spain	0.018	0.078	-0.476***	0.079
Sweden	-0.677***	0.086	-0.235**	0.078
Switzerland	-0.682***	0.096	-0.452***	0.088
**Age (Ref: 60–64)**				
50–54	-0.133**	0.050	-0.374***	0.047
55–59	-0.043	0.044	-0.171***	0.039
65–69	0.195***	0.043	0.107**	0.038
70–74	0.310***	0.045	0.156***	0.041
75–79	0.586***	0.053	0.251***	0.049
80–84	1.009***	0.059	0.334***	0.060
85–89	1.313***	0.074	0.364***	0.084
90–94	1.735***	0.123	0.290	0.170
**Women**	0.067**	0.026	0.458***	0.026
**Education (Ref: Medium)**				
Low	0.175***	0.031	0.163***	0.030
High	-0.274***	0.039	-0.297***	0.036
**Wave 5**	-0.460***	0.032	0.012	0.030
Constant	-1.952***	-0.079	-2.271***	-0.075
N	80,484	Pseudo R^2		0.034

Note: The dependent variable is a three-category variable that indicates if an individual achieved concordance (reference category), overestimated or underestimated his or her health. Coefficients are given in log odds, standard errors are clustered at the individual level, *p<0.05, **p<0.01, ***p<0.001

Table G. Multinomial logistic estimation for concordance between cognition measures (frail individuals are excluded from the sample)

	**Overestimating**	**SE**	**Underestimating**	**SE**
**Country (Ref: Slovenia)**				
Austria	-0.628***	0.070	-0.435***	0.056
Belgium	-0.446***	0.067	0.062	0.052
Czechia	-0.881***	0.071	0.241***	0.049
Denmark	-0.683***	0.081	-0.292***	0.061
Estonia	-0.647***	0.072	1.095***	0.047
France	-0.367***	0.066	0.327***	0.051
Germany	-0.499***	0.076	0.026	0.055
Hungary	-0.260**	0.097	0.426***	0.065
Italy	-0.293***	0.067	0.044	0.053
Luxembourg	-0.165	0.107	-0.496***	0.093
Netherlands	-0.674***	0.073	-0.531***	0.061
Poland	-0.005	0.107	0.239**	0.082
Portugal	-0.058	0.101	0.613***	0.073
Spain	-0.096	0.064	0.073	0.052
Sweden	-0.723***	0.078	0.227***	0.053
Switzerland	-0.828***	0.079	-0.389***	0.060
**Age (Ref: 60–64)**				
50–54	-0.244***	0.058	-0.254***	0.033
55–59	-0.198***	0.050	-0.107***	0.028
65–69	0.166***	0.047	0.134***	0.027
70–74	0.516***	0.047	0.343***	0.029
75–79	0.933***	0.047	0.443***	0.032
80–84	1.186***	0.051	0.364***	0.038
85–89	1.276***	0.064	0.133*	0.055
90–94	1.343***	0.112	-0.019	0.118
**Women**	-0.305***	0.026	0.078***	0.018
**Education (Ref: Medium)**				
Low	0.668***	0.032	0.264***	0.021
High	-0.429***	0.044	-0.314***	0.025
**Wave 5**	-0.114***	0.025	0.119***	0.016
Constant	-2.237***	-0.063	-1.690***	-0.048
N	103,816	Pseudo R-squared		0.058

Note: The dependent variable is a three-category variable that indicates if an individual achieved concordance (reference category), overestimated or underestimated his or her health. Coefficients are given in log odds, standard errors are clustered at the individual level, *p<0.05, **p<0.01, ***p<0.001

Table H. Multinomial logistic estimation for concordance between mobility measures (based on Wave 5 only)

	Overestimating	SE	Underestimating	SE
**Country (Ref: Slovenia)**				
Austria	-0.225**	0.085	-0.088	0.078
Belgium	-0.339***	0.082	0.141	0.073
Czechia	0.123	0.076	-0.041	0.074
Denmark	-0.848***	0.100	-0.246**	0.082
Estonia	-0.027	0.077	0.112	0.072
France	-0.267**	0.083	-0.157*	0.078
Germany	-0.386***	0.084	0.161*	0.072
Italy	-0.001	0.079	-0.345***	0.080
Luxembourg	-0.205	0.112	0.150	0.097
Netherlands	-0.757***	0.094	-0.297***	0.082
Spain	0.077	0.074	-0.465***	0.077
Sweden	-0.738***	0.091	-0.249**	0.079
Switzerland	-0.514***	0.098	-0.409***	0.090
**Age (Ref: 60–64)**				
50–54	-0.168*	0.068	-0.347***	0.057
55–59	-0.071	0.058	-0.148**	0.048
65–69	0.150**	0.055	0.098*	0.045
70–74	0.276***	0.056	0.133**	0.047
75–79	0.552***	0.056	0.265***	0.050
80–84	0.934***	0.058	0.313***	0.056
85–89	1.158***	0.067	0.219**	0.074
90–94	1.444***	0.099	0.105	0.133
**Women**	0.065*	0.030	0.412***	0.028
**Education (Ref: Medium)**				
Low	0.260***	0.037	0.133***	0.033
High	-0.280***	0.046	-0.308***	0.039
Constant	-2.389***	-0.075	-2.203***	-0.069
N	60,233	Pseudo R^2		0.033

Note: The dependent variable is a three-category variable that indicates if an individual achieved concordance (reference category), overestimated or underestimated his or her health. Coefficients are given in log odds, standard errors are clustered at the individual level, *p<0.05, **p<0.01, ***p<0.001

Table I. Multinomial logistic estimation for concordance between cognition measures (based on Wave 5 only)

	**Overestimating**	**SE**	**Underestimating**	**SE**
**Country (Ref: Slovenia)**				
Austria	-0.954***	-0.102	-0.198**	-0.066
Belgium	-0.323***	-0.083	0.078	-0.062
Czechia	-0.870***	-0.094	0.336***	-0.059
Denmark	-0.660***	-0.099	-0.276***	-0.069
Estonia	-0.694***	-0.098	1.242***	-0.057
France	-0.350***	-0.087	0.367***	-0.062
Germany	-0.394***	-0.087	-0.056	-0.062
Italy	-0.225**	-0.083	0.032	-0.063
Luxembourg	-0.103	-0.109	-0.397***	-0.092
Netherlands	-0.525***	-0.09	-0.496***	-0.07
Spain	-0.11	-0.078	-0.05	-0.061
Sweden	-0.554***	-0.091	0.187**	-0.062
Switzerland	-0.948***	-0.113	-0.214**	-0.072
**Age (Ref: 60–64)**				
50–54	-0.240**	-0.079	-0.232***	-0.044
55–59	-0.201**	-0.069	-0.105**	-0.037
65–69	0.176**	-0.063	0.116**	-0.035
70–74	0.438***	-0.063	0.312***	-0.036
75–79	0.859***	-0.062	0.444***	-0.039
80–84	1.023***	-0.066	0.336***	-0.045
85–89	1.132***	-0.077	0.114	-0.061
90–94	1.312***	-0.113	-0.144	-0.116
**Women**	-0.260***	-0.033	0.058**	-0.021
**Education (Ref: Medium)**				
Low	0.628***	-0.042	0.231***	-0.026
High	-0.510***	-0.058	-0.281***	-0.03
Constant	-2.326***	-0.08	-1.563***	-0.057
N	59,742	Pseudo R^2		0.059

Note: The dependent variable is a three-category variable that indicates if an individual achieved concordance (reference category), overestimated or underestimated his or her health. Coefficients are given in log odds, standard errors are clustered at the individual level, *p<0.05, **p<0.01, ***p<0.001

Table J. Multinomial logistic estimation for concordance between mobility measures (incl. indicator for employment)

	Overestimating	SE	Underestimating	SE
**Country (Ref: Slovenia)**				
Austria	-0.187*	0.08	-0.028	0.076
Belgium	-0.449***	0.078	0.124	0.071
Czechia	-0.043	0.074	-0.003	0.071
Denmark	-0.956***	0.094	-0.210**	0.080
Estonia	0.021	0.077	0.196**	0.073
France	-0.059	0.075	-0.194*	0.076
Germany	-0.268***	0.077	0.225**	0.071
Greece	0.066	0.089	-0.259**	0.099
Ireland	0.196	0.126	-0.082	0.148
Italy	0.236**	0.072	-0.238**	0.075
Luxembourg	-0.189	0.114	0.175	0.098
Netherlands	-0.897***	0.089	-0.224**	0.076
Poland	0.358***	0.092	0.288**	0.095
Spain	0.059	0.072	-0.351***	0.074
Sweden	-0.581***	0.082	-0.088	0.075
Switzerland	-0.569***	0.091	-0.331***	0.085
**Age (Ref: 60–64)**				
50–54	0.015	0.052	-0.151**	0.048
55–59	0.051	0.045	-0.036	0.040
65–69	0.114**	0.042	-0.009	0.037
70–74	0.242***	0.043	0.035	0.040
75–79	0.473***	0.050	0.120**	0.046
80–84	0.871***	0.055	0.180**	0.055
85–89	1.094***	0.065	0.113	0.073
90–94	1.338***	0.104	-0.008	0.139
**Women**	0.036	0.025	0.434***	0.025
**Education (Ref: Medium)**				
Low	0.158***	0.030	0.142***	0.028
High	-0.265***	0.038	-0.260***	0.035
**Wave 5**	-0.416***	0.030	0.042	0.029
**Employment**	-0.343***	0.040	-0.486***	0.037
Constant	-1.872***	0.075	-2.186***	-0.072
N	86,157	Pseudo R^2		0.035

Note: The dependent variable is a three-category variable that indicates if an individual achieved concordance (reference category), overestimated or underestimated his or her health. Coefficients are given in log odds, standard errors are clustered at the individual level, *p<0.05, **p<0.01, ***p<0.001

Table K. Multinomial logistic estimation for concordance between cognition measures (incl. indicator for employment)

	Overestimating	SE	Underestimating	SE
**Country (Ref: Slovenia)**				
Austria	-0.614***	0.066	-0.371***	0.053
Belgium	-0.405***	0.063	0.124*	0.049
Czechia	-0.836***	0.066	0.288***	0.047
Denmark	-0.623***	0.077	-0.192**	0.059
Estonia	-0.641***	0.067	1.153***	0.045
France	-0.322***	0.062	0.376***	0.049
Germany	-0.460***	0.072	0.077	0.053
Greece	-0.284**	0.087	0.502***	0.059
Ireland	-0.300***	0.063	0.076	0.051
Italy	-0.101	0.101	-0.426***	0.088
Luxembourg	-0.604***	0.070	-0.450***	0.058
Netherlands	-0.067	0.098	0.202**	0.077
Poland	-0.111	0.093	0.618***	0.068
Spain	-0.134*	0.059	0.108*	0.049
Sweden	-0.648***	0.074	0.329***	0.052
Switzerland	-0.794***	0.077	-0.284***	0.059
**Age (Ref: 60–64)**				
50–54	-0.131*	0.060	-0.072*	0.034
55–59	-0.107*	0.051	0.014	0.029
65–69	0.097*	0.046	0.021	0.027
70–74	0.450***	0.046	0.216***	0.028
75–79	0.809***	0.046	0.279***	0.031
80–84	1.013***	0.049	0.183***	0.035
85–89	1.101***	0.058	-0.084	0.049
90–94	1.163***	0.092	-0.174	0.094
**Women**	-0.302***	0.025	0.074***	0.017
**Education (Ref: Medium)**				
Low	0.633***	0.031	0.221***	0.020
High	-0.421***	0.043	-0.279***	0.024
**Wave 5**	-0.120***	0.024	0.122***	0.015
**Employment**	-0.282***	0.045	-0.410***	0.026
Constant	-2.142***	0.060	-1.574***	0.046
N	112,906	Pseudo R^2		0.057

Note: The dependent variable is a three-category variable that indicates if an individual achieved concordance (reference category), overestimated or underestimated his or her health. Coefficients are given in log odds, standard errors are clustered at the individual level, *p<0.05, **p<0.01, ***p<0.001

Table L. Multinomial logistic estimation for concordance between mobility measures (incl. indicator for children)

	**Overestimating**	**SE**	**Underestimating**	**SE**
**Country (Ref: Slovenia)**				
Austria	-0.220**	0.081	-0.046	0.076
Belgium	-0.438***	0.077	0.087	0.071
Czechia	-0.066	0.074	-0.056	0.071
Denmark	-0.973***	0.092	-0.303***	0.079
Estonia	-0.042	0.077	0.115	0.072
France	-0.101	0.075	-0.248**	0.076
Germany	-0.318***	0.077	0.161*	0.070
Greece	0.040	0.090	-0.282**	0.098
Ireland	0.138	0.125	-0.144	0.148
Italy	0.204**	0.073	-0.274***	0.075
Luxembourg	-0.210	0.112	0.156	0.097
Netherlands	-0.877***	0.087	-0.286***	0.076
Poland	0.388***	0.092	0.304**	0.095
Spain	0.020	0.073	-0.385***	0.074
Sweden	-0.637***	0.082	-0.193**	0.074
Switzerland	-0.631***	0.091	-0.423***	0.085
**Age (Ref: 60–64)**				
50–54	-0.143**	0.049	-0.349***	0.045
55–59	-0.052	0.043	-0.179***	0.038
65–69	0.192***	0.041	0.097**	0.037
70–74	0.330***	0.042	0.153***	0.039
75–79	0.574***	0.049	0.245***	0.046
80–84	0.971***	0.053	0.303***	0.054
85–89	1.177***	0.064	0.206**	0.072
90–94	1.449***	0.098	0.100	0.132
**Women**	0.057*	0.024	0.457***	0.025
**Education (Ref: Medium)**				
Low	0.181***	0.030	0.163***	0.028
High	-0.293***	0.038	-0.298***	0.035
**Wave 5**	-0.415***	0.030	0.028	0.029
**Children**	-0.247***	0.039	0.080	0.042
Constant	-1.728***	0.083	-2.344***	0.082
N	86,173	Pseudo R^2		0.033

Note: The dependent variable is a three-category variable that indicates if an individual achieved concordance (reference category), overestimated or underestimated his or her health. Coefficients are given in log odds, standard errors are clustered at the individual level, *p<0.05, **p<0.01, ***p<0.001

Table M. Multinomial logistic estimation for concordance between cognition measures (incl. indicator for children)

	**Overestimating**	**SE**	**Underestimating**	**SE**
**Country (Ref: Slovenia)**				
Austria	-0.627***	0.066	-0.387***	0.053
Belgium	-0.406***	0.062	0.087	0.049
Czechia	-0.844***	0.066	0.250***	0.047
Denmark	-0.657***	0.076	-0.263***	0.058
Estonia	-0.697***	0.067	1.077***	0.045
France	-0.352***	0.062	0.336***	0.049
Germany	-0.479***	0.071	0.03	0.053
Greece	-0.298***	0.086	0.497***	0.059
Ireland	-0.334***	0.063	0.038	0.051
Italy	-0.135	0.100	-0.426***	0.087
Luxembourg	-0.625***	0.069	-0.504***	0.058
Netherlands	-0.079	0.098	0.201**	0.077
Poland	-0.138	0.093	0.579***	0.068
Spain	-0.171**	0.059	0.061	0.049
Sweden	-0.689***	0.073	0.235***	0.051
Switzerland	-0.841***	0.076	-0.362***	0.058
**Age (Ref: 60–64)**				
50–54	-0.268***	0.057	-0.252***	0.033
55–59	-0.202***	0.049	-0.116***	0.027
65–69	0.162***	0.045	0.110***	0.026
70–74	0.527***	0.045	0.321***	0.028
75–79	0.888***	0.045	0.387***	0.030
80–84	1.092***	0.047	0.287***	0.035
85–89	1.172***	0.056	0.037	0.048
90–94	1.288***	0.086	-0.096	0.090
**Women**	-0.290***	0.025	0.091***	0.017
**Education (Ref: Medium)**				
Low	0.644***	0.031	0.240***	0.020
High	-0.446***	0.043	-0.306***	0.024
**Wave 5**	-0.130***	0.024	0.116***	0.015
**Children**	-0.230***	0.039	0.021	0.029
Constant	-1.984***	0.070	-1.672***	0.053
N	113,081	Pseudo R^2		0.056

Note: The dependent variable is a three-category variable that indicates if an individual achieved concordance (reference category), overestimated or underestimated his or her health. Coefficients are given in log odds, standard errors are clustered at the individual level, *p<0.05, **p<0.01, ***p<0.001

Table N. Multinomial logistic estimation for concordance between mobility measures (incl. indicator for marriage or registered partnership)

	Overestimating	SE	Underestimating	SE
**Country (Ref: Slovenia)**				
Austria	-0.215**	0.081	-0.046	0.076
Belgium	-0.421***	0.077	0.08	0.071
Czechia	-0.076	0.074	-0.061	0.071
Denmark	-0.982***	0.093	-0.310***	0.080
Estonia	-0.059	0.077	0.11	0.072
France	-0.105	0.075	-0.250***	0.076
Germany	-0.297***	0.077	0.157*	0.071
Greece	0.067	0.090	-0.315**	0.100
Ireland	0.157	0.125	-0.163	0.148
Italy	0.239**	0.073	-0.272***	0.075
Luxembourg	-0.195	0.112	0.157	0.097
Netherlands	-0.854***	0.087	-0.274***	0.076
Poland	0.400***	0.092	0.300**	0.096
Spain	0.053	0.073	-0.390***	0.074
Sweden	-0.635***	0.082	-0.188*	0.075
Switzerland	-0.621***	0.091	-0.446***	0.085
**Age (Ref: 60–64)**				
50–54	-0.138**	0.049	-0.358***	0.046
55–59	-0.044	0.042	-0.183***	0.038
65–69	0.190***	0.041	0.098**	0.036
70–74	0.322***	0.042	0.152***	0.039
75–79	0.545***	0.049	0.239***	0.046
80–84	0.936***	0.053	0.289***	0.054
85–89	1.129***	0.064	0.185*	0.073
90–94	1.391***	0.097	0.059	0.133
**Women**	0.020	0.025	0.444***	0.025
**Education (Ref: Medium)**				
Low	0.173***	0.030	0.157***	0.028
High	-0.293***	0.038	-0.300***	0.035
**Wave 5**	-0.411***	0.030	0.017	0.029
**Married**	-0.212***	0.027	-0.077**	0.027
Constant	-1.782***	0.079	-2.192***	0.076
N	85,781	Pseudo R^2		0.033

Note: The dependent variable is a three-category variable that indicates if an individual achieved concordance (reference category), overestimated or underestimated his or her health. Coefficients are given in log odds, standard errors are clustered at the individual level, *p<0.05, **p<0.01, ***p<0.001

Table O. Multinomial logistic estimation for concordance between cognition measures (incl. indicator for marriage or registered partnership)

	Overestimating	SE	Underestimating	SE
**Country (Ref: Slovenia)**				
Austria	-0.642***	0.066	-0.388***	0.053
Belgium	-0.410***	0.062	0.093	0.049
Czechia	-0.875***	0.066	0.251***	0.047
Denmark	-0.668***	0.076	-0.273***	0.058
Estonia	-0.719***	0.067	1.075***	0.045
France	-0.355***	0.062	0.337***	0.049
Germany	-0.472***	0.071	0.024	0.053
Greece	-0.303***	0.086	0.499***	0.059
Ireland	-0.315***	0.063	0.034	0.051
Italy	-0.125	0.101	-0.426***	0.087
Luxembourg	-0.619***	0.069	-0.507***	0.058
Netherlands	-0.070	0.098	0.200*	0.078
Poland	-0.124	0.093	0.588***	0.068
Spain	-0.153**	0.059	0.058	0.049
Sweden	-0.687***	0.073	0.238***	0.052
Switzerland	-0.832***	0.077	-0.360***	0.058
**Age (Ref: 60–64)**				
50–54	-0.280***	0.058	-0.259***	0.033
55–59	-0.197***	0.049	-0.119***	0.028
65–69	0.161***	0.045	0.112***	0.026
70–74	0.520***	0.044	0.322***	0.028
75–79	0.864***	0.045	0.387***	0.030
80–84	1.056***	0.047	0.288***	0.035
85–89	1.117***	0.057	0.033	0.048
90–94	1.200***	0.086	-0.099	0.090
**Women**	-0.337***	0.025	0.089***	0.017
**Education (Ref: Medium)**				
Low	0.639***	0.031	0.237***	0.020
High	-0.442***	0.043	-0.308***	0.024
**Wave 5**	-0.129***	0.024	0.117***	0.015
**Married**	-0.216***	0.027	-0.003	0.019
Constant	-2.007***	0.064	-1.649***	0.049
N	112,713	Pseudo R^2		0.056

Note: The dependent variable is a three-category variable that indicates if an individual achieved concordance (reference category), overestimated or underestimated his or her health. Coefficients are given in log odds, standard errors are clustered at the individual level, *p<0.05, **p<0.01, ***p<0.001

Table P. Multinomial logistic estimation for concordance between mobility measures (incl. interaction effect)

	Overestimating	SE	Underestimating	SE
**Country (Ref: Slovenia)**				
Austria	-0.195*	0.080	-0.049	0.075
Belgium	-0.420***	0.077	0.087	0.071
Czechia	-0.059	0.074	-0.049	0.071
Denmark	-0.965***	0.092	-0.309***	0.079
Estonia	-0.027	0.077	0.116	0.072
France	-0.084	0.075	-0.247**	0.075
Germany	-0.300***	0.076	0.162*	0.070
Greece	0.046	0.089	-0.299**	0.098
Ireland	0.168	0.125	-0.150	0.148
Italy	0.222**	0.072	-0.276***	0.075
Luxembourg	-0.195	0.112	0.151	0.097
Netherlands	-0.863***	0.087	-0.283***	0.076
Poland	0.395***	0.092	0.305**	0.095
Spain	0.037	0.072	-0.398***	0.074
Sweden	-0.632***	0.082	-0.192**	0.074
Switzerland	-0.607***	0.090	-0.430***	0.085
**Age (Ref: 60–64)**				
50–54	-0.132**	0.048	-0.356***	0.045
55–59	-0.048	0.042	-0.179***	0.038
65–69	0.193***	0.041	0.099**	0.036
70–74	0.333***	0.042	0.156***	0.039
75–79	0.568***	0.049	0.245***	0.045
80–84	0.975***	0.053	0.300***	0.054
85–89	1.197***	0.063	0.206**	0.072
90–94	1.485***	0.096	0.088	0.132
**Women**	0.029	0.041	0.388***	0.039
**Education (Ref: Medium)**				
Low	0.147***	0.042	0.094*	0.045
High	-0.272***	0.053	-0.378***	0.054
**Interaction Effects**				
Low x Women	0.061	0.054	0.109*	0.054
High x Women	-0.040	0.075	0.130	0.069
**Wave 5**	-0.414***	0.030	0.028	0.029
Constant	-1.953***	0.077	-2.228***	0.074
N	86,819	Pseudo R^2		0.033

Note: The dependent variable is a three-category variable that indicates if an individual achieved concordance (reference category), overestimated or underestimated his or her health. Coefficients are given in log odds, standard errors are clustered at the individual level, *p<0.05, **p<0.01, ***p<0.001

Table Q. Multinomial logistic estimation for concordance between cognition measures (incl. interaction effect)

	Overestimating	SE	Underestimating	SE
**Country (Ref: Slovenia)**				
Austria	-0.613***	0.066	-0.385***	0.053
Belgium	-0.380***	0.062	0.096	0.049
Czechia	-0.844***	0.066	0.256***	0.047
Denmark	-0.653***	0.076	-0.264***	0.058
Estonia	-0.672***	0.067	1.082***	0.045
France	-0.333***	0.061	0.334***	0.048
Germany	-0.473***	0.071	0.032	0.052
Hungary	-0.288***	0.086	0.495***	0.059
Italy	-0.312***	0.062	0.041	0.051
Luxembourg	-0.124	0.100	-0.427***	0.087
Netherlands	-0.616***	0.069	-0.496***	0.058
Poland	-0.068	0.098	0.204**	0.077
Portugal	-0.120	0.093	0.588***	0.068
Spain	-0.151*	0.059	0.064	0.049
Sweden	-0.670***	0.073	0.241***	0.051
Switzerland	-0.821***	0.076	-0.363***	0.058
**Age (Ref: 60–64)**				
50–54	-0.255***	0.056	-0.247***	0.032
55–59	-0.195***	0.049	-0.114***	0.027
65–69	0.160***	0.045	0.111***	0.026
70–74	0.524***	0.044	0.320***	0.028
75–79	0.882***	0.045	0.385***	0.030
80–84	1.090***	0.047	0.286***	0.035
85–89	1.175***	0.056	0.030	0.048
90–94	1.285***	0.085	-0.104	0.089
**Women**	-0.465***	0.046	0.020	0.027
**Education (Ref: Medium)**				
Low	0.501***	0.041	0.165***	0.030
High	-0.483***	0.055	-0.362***	0.035
**Interaction Effects**				
Low x Women	0.285***	0.056	0.128***	0.037
High x Women	0.071	0.087	0.098*	0.048
**Wave 5**	-0.126***	0.024	0.116***	0.015
Constant	-2.128***	0.061	-1.616***	0.047
N	113,812	Pseudo R^2		0.056

Note: The dependent variable is a three-category variable that indicates if an individual achieved concordance (reference category), overestimated or underestimated his or her health. Coefficients are given in log odds, standard errors are clustered at the individual level, *p<0.05, **p<0.01, ***p<0.001

Table R. Multinomial logistic estimation for concordance between mobility measures (incl. learning effect)

	**Overestimating**	**SE**	**Underestimating**	**SE**
**Country (Ref: Slovenia)**				
Austria	-0.153	0.080	-0.069	0.076
Belgium	-0.342***	0.078	0.046	0.071
Czechia	-0.004	0.074	-0.079	0.071
Denmark	-0.877***	0.093	-0.349***	0.080
Estonia	-0.032	0.077	0.112	0.072
France	-0.009	0.075	-0.284***	0.076
Germany	-0.245**	0.077	0.134	0.071
Greece	0.117	0.090	-0.335***	0.099
Ireland	0.237	0.126	-0.189	0.148
Italy	0.306***	0.073	-0.321***	0.076
Luxembourg	-0.193	0.112	0.149	0.097
Netherlands	-0.783***	0.088	-0.323***	0.077
Poland	0.469***	0.092	0.269**	0.096
Spain	0.092	0.073	-0.429***	0.074
Sweden	-0.560***	0.082	-0.231**	0.075
Switzerland	-0.537***	0.091	-0.465***	0.085
**Age (Ref: 60–64)**				
50–54	-0.169***	0.048	-0.337***	0.045
55–59	-0.063	0.042	-0.171***	0.038
65–69	0.197***	0.041	0.098**	0.036
70–74	0.342***	0.042	0.153***	0.039
75–79	0.585***	0.049	0.239***	0.045
80–84	0.947***	0.053	0.314***	0.054
85–89	1.132***	0.064	0.236**	0.073
90–94	1.418***	0.097	0.123	0.132
**Women**	0.057*	0.024	0.457***	0.024
**Education (Ref: Medium)**				
Low	0.183***	0.030	0.163***	0.028
High	-0.290***	0.038	-0.299***	0.035
**Wave 5**	-0.337***	0.032	-0.006	0.031
**Learning effect**	-0.311***	0.043	0.115***	0.035
Constant	-2.033***	0.075	-2.238***	0.073
N	86,819	Pseudo R^2		0.033

Note: The dependent variable is a three-category variable that indicates if an individual achieved concordance (reference category), overestimated or underestimated his or her health. Coefficients are given in log odds, standard errors are clustered at the individual level, *p<0.05, **p<0.01, ***p<0.001

Table S. Multinomial logistic estimation for concordance between cognition measures (incl. learning effect)

	**Overestimating**	**SE**	**Underestimating**	**SE**
**Country (Ref: Slovenia)**				
Austria	-0.575***	0.066	-0.452***	0.053
Belgium	-0.332***	0.063	0.000	0.049
Czechia	-0.820***	0.066	0.203***	0.047
Denmark	-0.593***	0.076	-0.349***	0.058
Estonia	-0.683***	0.067	1.047***	0.045
France	-0.281***	0.062	0.231***	0.049
Germany	-0.467***	0.072	0.043	0.052
Hungary	-0.326***	0.086	0.560***	0.060
Italy	-0.268***	0.063	-0.046	0.051
Luxembourg	-0.203*	0.101	-0.254**	0.088
Netherlands	-0.562***	0.070	-0.585***	0.058
Poland	0.074	0.100	-0.050	0.078
Portugal	-0.169	0.093	0.644***	0.068
Spain	-0.137*	0.059	0.027	0.049
Sweden	-0.636***	0.074	0.186***	0.051
Switzerland	-0.764***	0.077	-0.460***	0.058
**Age (Ref: 60–64)**	-0.319***	0.057	-0.141***	0.033
50–54	-0.205***	0.049	-0.098***	0.027
55–59	0.165***	0.045	0.107***	0.026
65–69	0.534***	0.044	0.311***	0.028
70–74	0.893***	0.045	0.376***	0.030
75–79	1.105***	0.047	0.274***	0.035
80–84	1.193***	0.056	0.015	0.048
85–89	1.307***	0.086	-0.114	0.090
90–94	-0.319***	0.057	-0.141***	0.033
**Women**	-0.287***	0.025	0.085***	0.017
**Education (Ref: Medium)**				
Low	0.643***	0.031	0.238***	0.020
High	-0.447***	0.043	-0.308***	0.024
**Wave 5**	-0.084***	0.025	0.003	0.016
**Learning effect**	-0.193***	0.027	0.337***	0.018
Constant	-2.165***	0.059	-1.722***	0.046
N	113,812	Pseudo R^2		0.058

Note: The dependent variable is a three-category variable that indicates if an individual achieved concordance (reference category), overestimated or underestimated his or her health. Coefficients are given in log odds, standard errors are clustered at the individual level, *p<0.05, **p<0.01, ***p<0.001

References Appendix

1. Fried LP, Tangen CM, Walston J, Newman AB, Hirsch C, Gottdiener J, et al. Frailty in older adults: evidence for a phenotype. J Gerontol A Biol Sci Med Sci. 2001;56A(3):M146-56.
2. Santos-Eggimann B, Cuénoud P, Spagnoli J, Junod J. Prevalence of frailty in middle-aged and older community-dwelling Europeans living in 10 countries. Journals Gerontol - Ser A Biol Sci Med Sci. 2009;64A(6):675–81.
