# Supplementary figures and images for: Reporting biases in self-assessed physical and cognitive health status of older Europeans
Source: PLoS One. 2019 Oct 8;14(10):e0223526. doi: 10.1371/journal.pone.0223526 (PMC6783110; doi:10.1371/journal.pone.0223526)

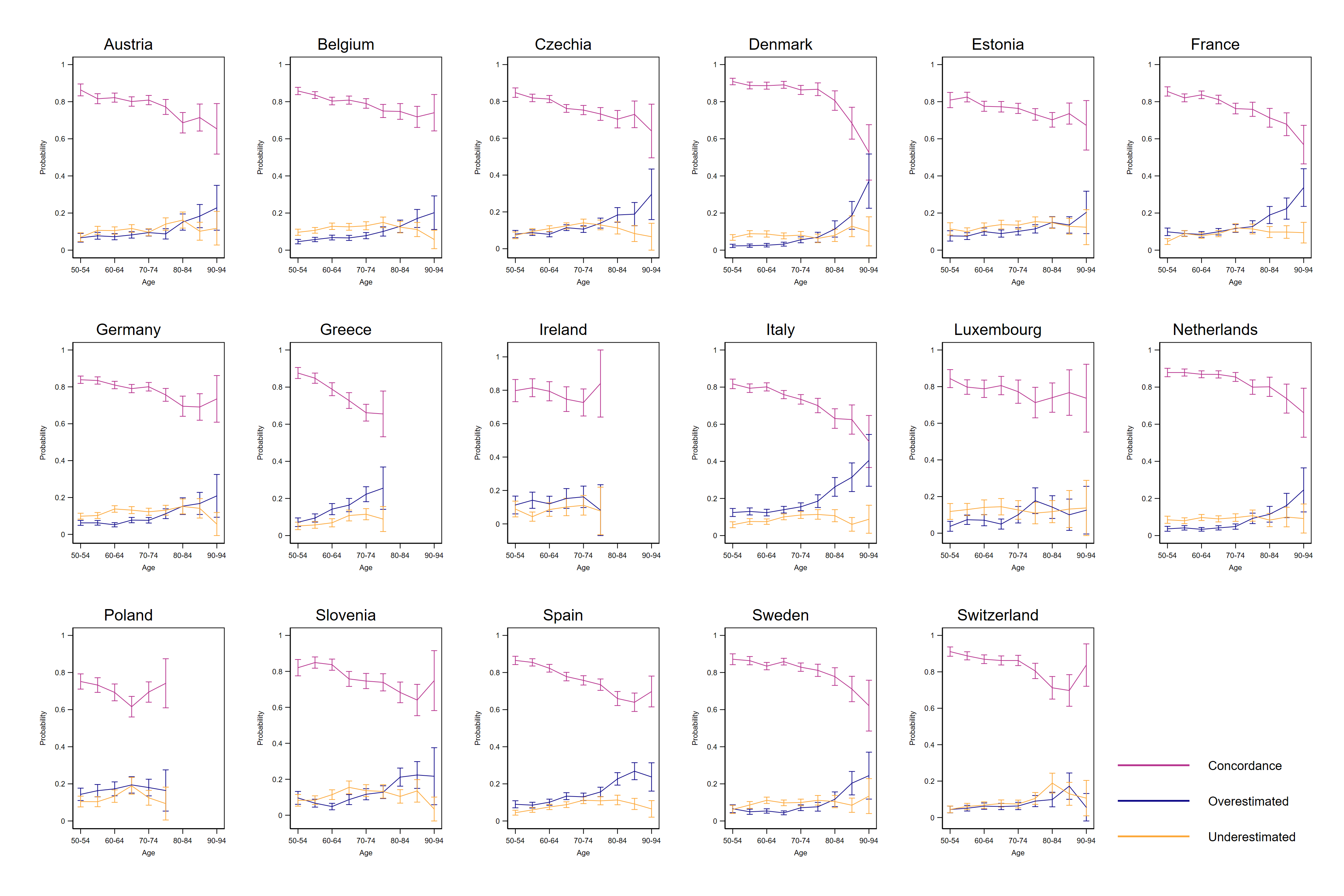

Supplement: S1 Fig — (TIF) [file pone.0223526.s002.tif]

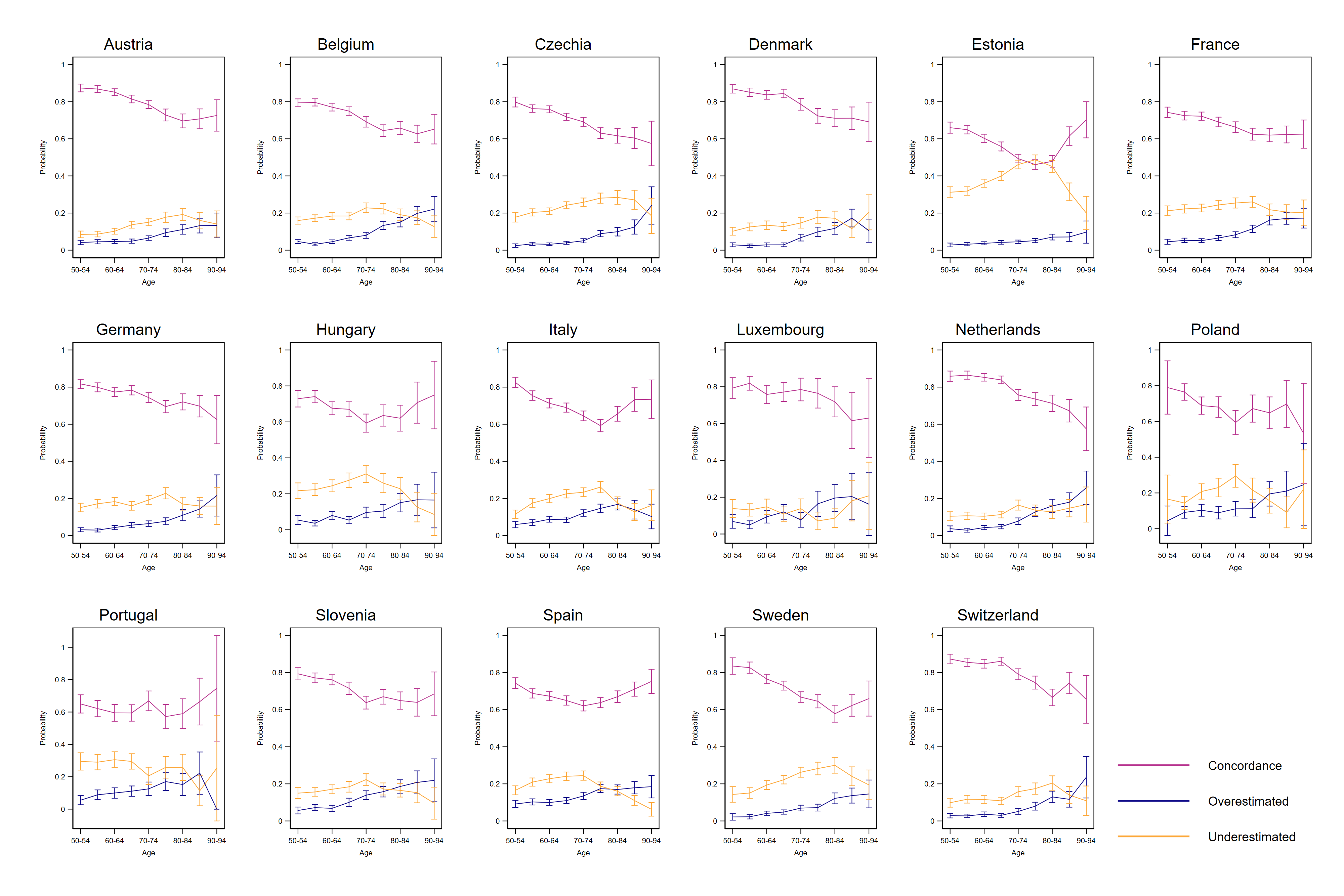

Supplement: S2 Fig — (TIF) [file pone.0223526.s003.tif]
